# Energy landscapes of Aβ monomers are sculpted in accordance with Ostwald’s rule of stages

**DOI:** 10.1126/sciadv.add6921

**Published:** 2023-03-22

**Authors:** Debayan Chakraborty, John E. Straub, D. Thirumalai

**Affiliations:** ^1^ Department of Chemistry, The University of Texas at Austin, 105 E 24th Street, Stop A5300, Austin TX 78712, USA.; ^2^ Department of Chemistry, Boston University, MA 022155, USA.

## Abstract

The transition from a disordered to an assembly-competent monomeric state (N*) in amyloidogenic sequences is a crucial event in the aggregation cascade. Using a well-calibrated model for intrinsically disordered proteins (IDPs), we show that the N* states, which bear considerable resemblance to the polymorphic fibril structures found in experiments, not only appear as excitations in the free energy landscapes of Aβ40 and Aβ42, but also initiate the aggregation cascade. For Aβ42, the transitions to the different N* states are in accord with Ostwald’s rule of stages, with the least stable structures forming ahead of thermodynamically favored ones. The Aβ40 and Aβ42 monomer landscapes exhibit different extents of local frustration, which we show have profound implications in dictating subsequent self-assembly. Using kinetic transition networks, we illustrate that the most favored dimerization routes proceed via N* states. We argue that Ostwald’s rule also holds for the aggregation of fused in sarcoma and polyglutamine proteins.

## Teaser

Amyloid beta monomers adopt transient fibrillar order following Ostwald's rule of stages.

## INTRODUCTION

The accumulation of insoluble plaques and neurofibrillary tangles inside different parts of the central nervous system is a hallmark of Alzheimer’s disease (AD), one of the well-known proteinopathies (*1*–*3*). The amyloid plaques have a characteristic cross-β architecture and are primarily composed of β-amyloid (Aβ) peptides of length 39 to 43 residues (*1*). These peptides are produced in vivo by the proteolytic cleavage of the nearly 770-residue β-amyloid precursor protein (APP) by γ-secretases (*4*), with Aβ40 and Aβ42 being the most common cleavage products. Despite having globally similar topologies, Aβ fibrils exhibit diverse structural polymorphism (*5*–*7*). The specific structure that is populated could depend on the differences in the fibril growth conditions (*8*) and the preferential hydration of certain polymorphs (*9*). Although Aβ fibrils were considered to be the key players in AD etiology (*10*, *11*), recent works suggest that the oligomers (*12*–*14*) formed early along the aggregation cascade could be the real culprits. Other neurodegenerative disorders, such as Parkinson’s disease (PD) and Huntington’s disease, which also result from aberrant protein aggregation, may share certain common themes with AD (*15*, *16*), including mechanisms of fibril formation, polymorphic fibril morphologies, and the association of cytotoxicity with oligomers. To uncover the general principles, a microscopic understanding of the key steps connecting the monomer to the fibril state is necessary (*17*).

The first step in the aggregation cascade involves the conformational transitions of the Aβ monomer to an assembly-competent structure (henceforth referred to as the N* state), having some structural signatures of the fibril state. The N* states are excitations on the monomer free energy landscape (*18*–*21*). Because the N* states are only sparsely populated (typically less than 5%) under normal growth conditions, they can only be resolved by experiments with high spatial and temporal resolution (*22*, *23*). In this context, computer simulations are useful in directly detecting these “dark states.” The different N* states could self-assemble to form oligomers of different sizes. A critical nucleus once formed, serves as a template for protofibril formation, and it continues to grow through the deposition of monomers via a “dock-lock” mechanism (*17*). In this model of protein aggregation (*17*, *19*), the N* concept plays a central role. It not only accounts for the possibility of fibril polymorphism, but also provides a basis for understanding the aggregation propensities of different peptide sequences (*21*, *24*).

The Aβ40 and Aβ42 sequences, which are the major isoforms implicated in AD, behave similar to intrinsically disordered proteins (IDPs) in their monomeric forms (*23*, *25*), which is manifested by a lack of persistent secondary structures. Hence, a microscopic characterization of their conformational ensembles is a challenging task. Toward this end, computer simulations (*26*–*28*) have provided important insights into the structural ensembles of Aβ monomers, although the precise details and the corresponding ensemble averages strongly depend on the force-field and the sampling strategy. Using the self-organized polymer model for IDPs (SOP-IDP), we showed in a recent study (*21*) that ensemble averages cannot be used to distinguish between the aggregation propensities of Aβ40 and Aβ42 because much of the statistical weight is dominated by random coil (RC)–like structures. In this sense, Aβ40 and Aβ42 behave similar to the Flory RC, with *R*_g_ ∼ *a*_0_*N^ν^* (ν ≈ 0.6), where *N* is the number of amino acids. It is only when the population of the different fibril-like states were identified using clustering techniques and geometric order parameters, the nearly one order of magnitude difference (*29*, *30*) between the aggregation rates of Aβ40 and Aβ42 could be rationalized. While some studies (*30*, *31*) have quantified the rates for the individual steps along the aggregation cascade (primary nucleation, secondary nucleation, fibril elongation, and fibril fragmentation), for our purposes, aggregation rate simply corresponds to the mean first passage time (MFPT), τ_fib_, for fibril assembly, starting from the lowest-energy monomer conformation. This definition was introduced by Li *et al.* (*19*) in the context of lattice simulations and naturally subsumes the primary nucleation and fibril extension rates. Our previous work (*21*) underscores the importance of quantitatively characterizing the N* states present within the monomer conformational ensemble (MCE) of assembly-competent IDPs to provide a microscopic basis for protein aggregation (*21*, *24*). We showed that for Aβ42 at least two N* states could exist, which naturally accounted for the different fibril topologies identified in experiments. On the basis of the notable analogy between crystallization and aggregation, we also asserted that different fibril polymorphs must appear according to Ostwald’s rule of stages.

In this work, we convincingly show that our earlier assertion indeed holds, by characterizing the free energy landscapes of Aβ40 and Aβ42 from extensive kinetic simulations based on the SOP-IDP model. As we describe below, the energy landscapes are sculpted in accordance with Ostwald’s rule of stages, with less stable N* states appearing before thermodynamically favored ones. In our earlier work (*21*), we described the differences in the aggregation behavior of Aβ40 and Aβ42 from a thermodynamic standpoint. Here, we illustrate that the additional subtleties (of kinetic origin) that could dictate different steps along the assembly cascade, may be identified from the contrasting topological features of the monomer energy landscapes.

For both peptides, the free energy ground state comprises an ensemble of disordered RC structures, rationalizing why many of the thermodynamic ensemble averages are reminiscent of RCs. A diverse range of N* states are encoded as excitations on the energy landscape, and they exhibit the key structural features found in different Aβ40 and Aβ42 polymorphs, such as the U-bend (*32*) and the S-bend motifs (*33*). Despite having similar topographies, the landscapes are associated with different extents of local frustration, particularly in regions where the probability to find the N* structures is the maximum. This finding has important implications in the intramolecular diffusivity of Aβ monomers and, subsequently, their ability to coalesce with other binding partners. The dynamics of Aβ monomers are hierarchically organized, with relaxation time scales in the submicrosecond regime, in agreement with recent experiments (*34*, *35*).

Within the premise of the N* theory (*19*), the fibril formation time scales, τ_fib_, only depend exponentially on the population of fibril-like conformations within the conformational ensemble. By definition, τ_fib_, encompasses the time scales associated with the individual steps along the aggregation cascade. Hence, it is natural to ask if Aβ40 and Aβ42 exhibit distinct behaviors in terms of individual rate constants? Here, we probe the kinetics of the very first step, which is the transition from the RC ground state to assembly-competent N* conformations. We find that the transitions between the RC configurations and the N* states are faster in Aβ42, suggesting that it is kinetically more predisposed to aggregate compared to Aβ40. Once formed, the N* state also survives longer for Aβ42 due to the enhanced frustration on the landscape. Although there are multiple pathways to dimer formation, the most productive route is the one when both the monomers are in the N* state. Together, it means that Aβ42 has a higher propensity to self-assemble and form oligomeric structures through efficient templating by the N* states.

The order of transitions to the different N* states in Aβ42 is in accord with Ostwald’s rule of stages, with the thermodynamically less favored U-bend structure appearing before the S-bend conformation. Thus, thermodynamic stability and fibril formation rates are inversely correlated, which we argue also prevails in the formation of fibrils in fused in sarcoma (FUS) and the associated variants, and in polyglutamine peptides.

## RESULTS

### Free energy landscapes as transition disconnectivity graphs

The conformational spaces of the Aβ monomers were partitioned into distinct clusters (free energy minima) based on the distribution of reciprocal interatomic distances (DRID) metric. As described in earlier work (*36*), the DRID-based metric preserves the kinetic distances among different minima. The optimal number of clusters for Aβ40 and Aβ42 was identified using a knee-point analysis (see the Supplementary Materials and fig. S2). The effective free energy barriers between different minima were estimated using the min-cut procedure (*37*), which is based on the Ford and Fulkerson theorem (*38*), and exploits the isomorphism between a network representation of the conformational landscape and a graph with capacitated edges (see the Supplementary Materials for further details).

**Fig. 1. F1:**
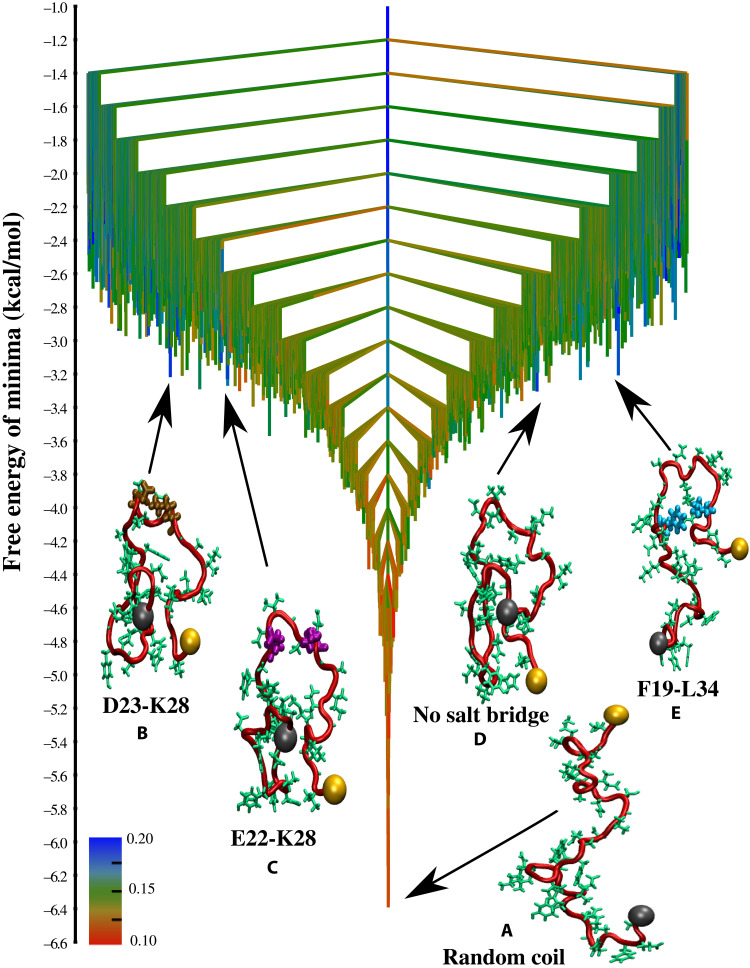
TRDG for Aβ40. The free energy landscape for the Aβ40 monomer at 298 K depicted in the form of a TRDG. The branches are colored according to $\chi_{U}^{\min}$, which is the minimum value of the overlap with respect to the experimental U-bend fibril structure, for a group of conformations constituting a free energy minimum (the scale quantifies the structural similarity). The N terminus of the peptide is shown as a gray sphere, and the C terminus is shown as an orange sphere. The key contacts within these structures are shown in different colors: D23-K28 salt-bridge (ochre), E22-K28 salt-bridge (purple), and F19-L34 contact (cyan). An ensemble of RC structures corresponds to the free energy global minimum (**A**). The landscape exhibits minimal frustration, suggesting that relaxation to the global minimum from other regions of the landscape occur efficiently. Some representative snapshots corresponding to the different N* states (free energy excitations that resemble the fibril structure) are also shown. (**B**) SLS topology stabilized by a VGSN turn and a D23-K28 salt-bridge. (**C**) SLS structure stabilized by a VGSN turn and a E22-K28 salt-bridge. (**D**) SLS structure lacking the E22/D23-K28 salt-bridge. (**E**) SLS structure with a contact between residues F19 and L34.

**Fig. 2. F2:**
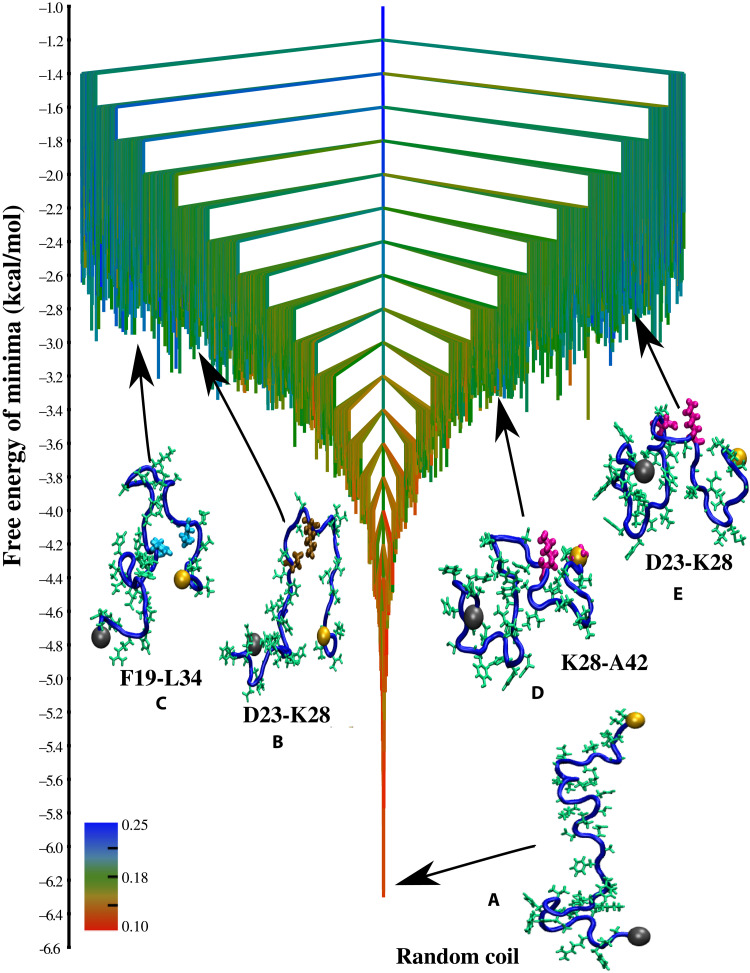
TRDG for Aβ42. The free energy landscape for the Aβ42 monomer computed at 298 K depicted in the form of a TRDG. The branches of the TRDG are color-coded according to either $\chi_{U}^{\min}$ or $\chi_{S}^{\min}$, whichever is greater for a given free energy minimum. Here, $\chi_{U}^{\min}$ is the minimum value of the overlap with respect to the U-bend fibril, and $\chi_{S}^{\min}$ is the minimum value of the overlap with respect to the S-bend fibril for conformations within a free energy minimum. The N terminus of the peptide is shown as a gray sphere, and the C terminus is shown as an orange sphere. RC structures constitute the free energy global minimum (**A**), and the landscape appears minimally frustrated (similar to Aβ40). Some representative snapshots corresponding to the different N* states are also shown. (**B**) SLS-like structure consisting of a U-bend near the VGSN turn region and stabilized by a D23-K28 salt-bridge (shown in ochre). (**C**) SLS structure stabilized by a F19-L34 contact (shown in cyan). (**D**) S-bend motif stabilized by a K28-A42 contact (shown in magenta). (**E**) S-bend motif having the K28-A42 contact replaced by a D23-K28 salt-bridge.

The free energy landscapes of Aβ monomers at 298 K are depicted in the form of disconnectivity graphs (*37*, *39*). In contrast to other formulations that rely on low-dimensional projections onto predefined order parameters, a disconnectivity graph provides a faithful representation of the underlying kinetics. In this “tree” structure (*37*, *39*, *40*) (or equivalently the “kinetic transition network” representation) (*41*), the landscape is partitioned into disjoint free energy basins, such that minima within each basin are mutually accessible, whereas interbasin transitions only occur over longer observation time scales (see the Supplementary Materials for further details). In Figs. 1 and 2, we represent the landscapes in terms of transition disconnecitivity graphs (TRDGs), which depict the complex dynamics on the landscape directly, in terms of hops between discretized microstates, without any assumptions in the description of the underlying dynamics. Previously, we have used this convention to describe the dynamics of salt-bridge formation in Aβ peptides (*18*) and the effect of single-point mutations (*42*). Alternatively, the landscapes can also be depicted in terms of free energy disconnectivity graphs (FEDGs) (*37*, *40*), where the free energy barriers are derived from unimolecular rate constants. The FEDGs of Aβ40 and Aβ42 are included in the fig. S5. As is evident, the FEDGs capture the overall flatness of the energy landscape, a characteristic feature of many IDPs (*28*, *43*).

Each node (free energy minimum) in the TRDG (Figs. 1 and 2) corresponds to an ensemble of structures that undergo rapid interconversion through local fluctuations. The TRDG, therefore, is an effective representation of the structural heterogeneity of Aβ monomers (*21*) and the hierarchical organization of their conformational dynamics (*35*). Both features are known to mediate the early events along the aggregation cascade. The branches of the TRDGs (Figs. 1 and 2) are color-coded from red to blue to reflect the degree of structural similarity with respect to the experimental fibril structures (see captions in Figs. 1 and 2 for details).

For both Aβ40 and Aβ42, RCs devoid of any persistent structure represent the free energy ground state and appear at the bottom of the energy landscapes (Figs. 1 and 2). The branches near the bottom of the TRDGs are colored red, indicating that conformations within the lowest free energy minima do not exhibit any structural similarity with respect to the monomers within the fibril states. [Fig F1][Fig F2]Hence, RCs would largely determine the thermodynamic properties (in other words, the experimentally measurable observables) at low temperatures. It is therefore not surprising that previous experimental studies have established nearly negligible secondary structure propensities for Aβ40 and Aβ42 peptides (*25*) and practically identical ensemble averages for global chain dimensions (*34*).

The color of branches continuously changes from red to blue, with uphill excursions on the TRDGs. In other words, the essential signatures of the fibril-like order encoded within the free energy excitations (N* states) of the MCEs become apparent, despite the evident bias toward RC-like conformations at equilibrium. It is important to note that due to a fine partitioning of the configuration space, fibril-like conformations are scattered across various clusters in the TRDGs. In Figs. 1 and 2, we show snapshots corresponding to the lowest free minima, which exhibit the key structural elements of the fibril state. The N* conformations are only sparsely populated relative to the disordered state (with populations ≈ 5% or less) (*20*–*22*), and their structural details are described below.

#### Aβ40

Previous studies on Aβ monomers (*44*–*46*) and oligomers, (*47*) as well as the kinetics of fibril formation for a designed Aβ40 (D23-K28) lactam construct (*48*) underscore the key role of the D23-K28 salt-bridge in amyloid aggregation. In earlier works (*18*, *49*), we have probed the dynamics of the D23-K28 salt-bridge using atomically detailed simulations and shown that it could substantially affect the kinetics of fibril formation. Conformations within free energy minimum B (Fig. 1B) exhibit these contacts and form the strand-loop-strand (SLS) structure, which forms the repeating unit in the U-bend Aβ40 fibril (*50*). Structures within minimum C (Fig. 1C) also exhibit the same SLS topology but consist of a salt-bridge between residues E22 and K28. Statistical analysis from our earlier study showed that the D23-K28 salt-bridge is only marginally favored compared to E22-K28 (*21*). Here, we find that the corresponding free energy minima are approximately isoenergetic. In addition to minima B and C (Fig. 1, B and C), where nearly all the constituent structures have the E22/D23-K28 salt-bridges, we also find minima, which are structurally more heterogeneous. For instance, in minimum D (Fig. 1D), all the structures exhibit the SLS topology, but the E22/D23-K28 salt-bridges are absent in most of them. The appearance of these structures on the free energy landscape hints at a scenario where the Aβ40 monomer could adopt the fibril-like topologies at the monomer level, with the D23-K28 contact appearing late during the aggregation cascade. This possibility was raised recently (*51*), where using solid-state nuclear magnetic resonance (NMR) spectroscopy, the authors speculated that the D23-K28 salt-bridge does not form at the monomer or the early oligomer stage.

In addition to the E22/D23-K28 salt-bridges, we also find many SLS topologies exhibiting a contact between the F19 and L34 residues (Fig. 1E). Previous studies have shown that the perturbations of the F19-L34 can completely abrogate the cytotoxicity of Aβ40 fibrils (*52*, *53*), without inducing substantial distortions in the fibril structure. Experiments based on solid-state NMR spectroscopy (*52*–*54*) indicate that unlike the D23-K28 salt-bridge, which may be absent in early assembly intermediates, the F19-L34 contact persists throughout the aggregation cascade.

#### Aβ42

Similar to Aβ40, we also find a diverse range of excited states on the free energy landscape of Aβ42, exhibiting fibril-like morphologies. Free energy minimum B (Fig. 2B) consists of structures having the canonical U-bend (SLS topology) and a salt-bridge between residues D23 and K28. These structures resemble the repeating units found in some Aβ42 polymorphs (*55*). Just like Aβ40, we also identify free energy minima in the intermediate sections of the TRDG having a SLS topology stabilized by a F19-L34 contact (Fig. 2C).

Besides the U-bend polymorph, Aβ42 also forms fibril structures in which the building block is a S-bend motif. Recent NMR (*33*, *56*) and cryo–electron microscopy (cryo-EM) (*57*) experiments have shown that the S-bend results from enhanced structural ordering near the C terminus of Aβ42, and it is characterized by a hydrophobic contact between residues K28 and A42. The exclusive formation of the S-bend motif in the case of Aβ42 could be linked to its higher aggregation propensity as compared to Aβ40 (*58*). All the structures within the free energy minimum D (Fig. 2D) have the characteristic features of the S-bend motif and exhibit a stable contact between residues K28 and A42. The S-bend structures within minimum E (Fig. 2E) lack the K28-A42 contact and are substantially destabilized, suggesting that hydrophobic interactions between the terminal residues are critical for maintaining the complex topology. In agreement with our recent study (*21*), where we estimated the relative populations of different Aβ42 conformational ensembles based on structural clustering, we find that the lowest free energy minimum consisting of S-bend topologies (Fig. 2D) appears about 0.2 kcal/mol lower (table S3) in the TRDG than the corresponding minimum for U-bend structures (Fig. 2B). To estimate the cumulative populations of the different N* states, all the fibril-like conformations (as determined from a structural order parameter) scattered across the different free energy minima must be taken into account.

#### Relaxation time scales

We estimated the time scales associated with the relaxation of the different states, corresponding to the nodes in the TRDGs, to their equilibrium distributions. We assume that the kinetics may be described in terms of a discrete time Markov chain (*59*, *60*). For a given vector, **P**(**t**), whose elements are the probabilities of finding the system in different states at time *t*, the following holds for Markovian dynamics

P(t+τ)=T(τ)P(t) (1)

In Eq. 1, **T**(τ) is the transition matrix, whose elements *T_ij_*s are the probabilities of finding the system in state *j* at time τ when it was in state *i* at time *t*. The highest eigenvalue of *T*(τ) is exactly 1, and the corresponding eigenvector represents the populations of the different states at thermal equilibrium. The other eigenvalues, λ*_i_*, are related to the implied relaxation time scales, *t_i_*, in the following way

$$t_{i}=\frac{-\tau }{\ln|\lambda_{i}(\tau )|}$$ (2)

We find that for both Aβ40 and Aβ42, the implied time scales, *t_i_*, are largely independent of the lag time, τ (see fig. S7). This convergence implies that the dynamics are approximately Markovian and attests to the accuracy of the state-space discretization.

**Fig. 3.**
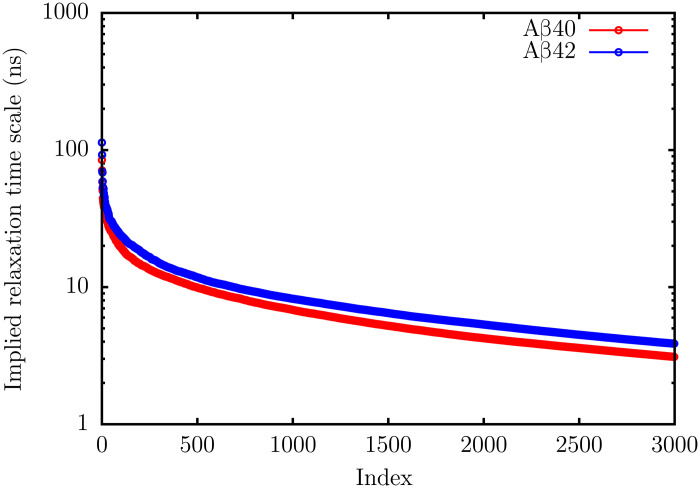
Relaxation time scales. The top 3000 relaxation time scales, *t_i_*, for Aβ40 (red) and Aβ42 (blue) estimated using Eq. 2. As is evident, there are no relaxation processes in the microsecond to millisecond regime. The *t_i_*s start to plateau at around 3 ns, and these time scales are associated with the fastest processes (local deformation modes of the peptides) on the energy landscapes.

Neither Aβ40 nor Aβ42 exhibits any relaxation process in the microsecond to millisecond regime (Fig. 3), in agreement with the findings of recent nanosecond fluorescence spectroscopy (nFCS) experiments (*34*). The slowest relaxation time scale for Aβ40 is around 85 ns, while for Aβ42, it is around 113 ns. The *t_i_* spectra for both peptides converge on time scales of ≈3 ns. These are the fastest relaxation processes that can be captured by our state-space discretization and correspond to the local deformation modes of the peptides.

#### Implication of landscape frustration

**Fig. 4.**
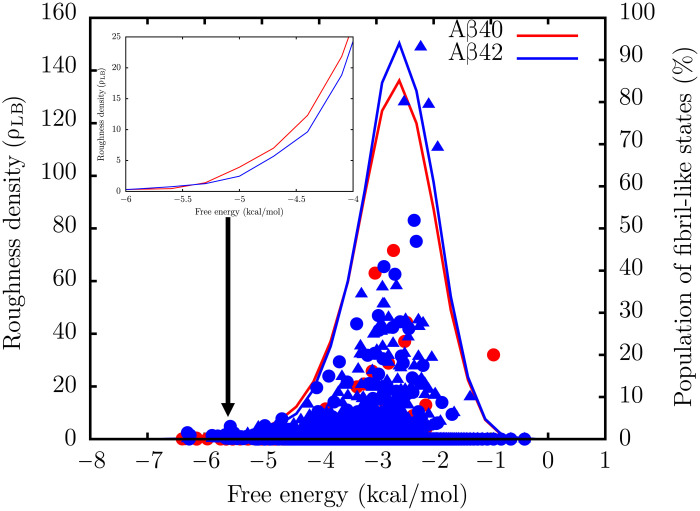
Variation of landscape roughness density with free energy. The solid lines show the variation of the landscape roughness density, ρ_LB_, with free energy. The roughness density was computed from the TRDGs using the scheme of Levy and Becker (*61*). The symbols denote the percentages of fibril-like structures within the free energy minima at each energy level: Aβ40 (red circles), Aβ42 U-bend (blue circles), and Aβ42 S-bend (blue triangles). Inset: The variation of the landscape roughness for Aβ40 (red) and Aβ42 (blue) close to the free energy of the ground state.

The population of fibril-like conformations (N* states) within each free energy minimum was computed by using a stringent geometric criterion (*21*). The structural order parameter, $\chi_{\mathrm{fib}}^{i}(m)$, determines the similarity between a conformation *i* within free energy minimum, *m*, and a monomer unit in the experimental fibril structure

$$\chi_{\mathrm{fib}}^{i}(m)=\frac{1}{N_{p}}\sum_{j=1}^{N_{p}}H[d-|r_{j}^{i}(m)-r_{j}^{0}|]$$ (3)

where *H* is the Heaviside step function, *N*_p_ is the total number of pairwise distances, and $r_{j}^{0}$ denotes the distance between the *j*-th pair of beads in the reference structure. For Aβ40, we use the brain-derived fibril structure [Protein Data Bank (PDB) ID: 2M4J] (*32*) as the reference, while for Aβ42, we use the experimental fibril structures corresponding to the U-bend (PDB ID: 2BEG) (*55*) and the S-bend (PDB ID: 2NAO) (*33*) morphologies as the reference states. The conformation *i* is aggregation prone (assembly-competent) if $\chi_{\mathrm{fib}}^{i}(m)\geq \chi_{c}$. We set χ*_c_* = 0.30 for both Aβ40 and Aβ42. The population of fibril-like conformations within each free energy minimum (Fig. 4) is then simply the percentage of constituent structures for which χ_fib_ ≥ χ*_c_*.

To quantify the local frustration on the free energy landscape, we computed the roughness density, ρ_LB_, from the TRDGs using the formalism of Levy and Becker (*61*) (see the Supplementary Materials for details). We find that for both Aβ40 and Aβ42, ρ_LB_ is substantial only over a narrow range of free energies, implying that similar to other IDPs (*43*, *62*), the energy landscapes of Aβ40 and Aβ42 exhibit a rather shallow funnel (*61*). This scenario is in contrast to fast-folding globular proteins, where the slope of the energy landscape tends to be quite steep (*43*).

As is evident from Fig. 4, ρ_LB_, is somewhat higher for the Aβ42 peptide in regions of the landscape where the propensity to find N* states (especially the S-bend motif) is maximal. An immediate implication of this result is that for Aβ42, the landscape in the vicinity of N* states is more undulated compared to Aβ40. Therefore, the aggregation-prone structures have a longer time to form an encounter complex via self-association. Our findings recapitulate the key features of the intramolecular diffusion model for Aβ aggregation kinetics, proposed in a previous work (*63*). We also estimated the frustration metric for Aβ40 and Aβ42 as a function of temperature using the formulation described by Wales and colleagues (*64*). This metric describes how efficiently a system relaxes to the global minimum and has been used to characterize the contrasting dynamics of structure seekers, glass formers, and biomolecules. We find that the energy landscape of Aβ42 is more frustrated than Aβ40, particularly at low temperatures (fig. S8), thus corroborating our inferences from the roughness density analysis.

Aβ42 exhibits a smaller ρ_LB_ close to the free energy ground state (disordered basin) relative to Aβ40 (Fig. 4, inset). This implies that excitations out of the RC state to fibril-like structures are likely to be more favorable in Aβ42.

#### Kinetics of interconversion

The free energy landscapes constitute transition networks (*41*, *65*), where the minima represent the nodes, and the intervening barriers correspond to the edges. Various kinetic observables of interest, such as rate constants (or equivalently MFPTs) can be extracted from these networks. Using a graph transformation method (*66*), we estimated the MFPTs corresponding to the transitions between the RC ground state and various excited states that are rich in fibril-like conformations. For the RC → U-bend transition in Aβ40, we estimate a MFPT of ≈32 μs. The U-bend structures relax rather quickly to the ground state, with a MFPT of ≈7 ns. The transition to the U-bend structure in Aβ42 from the RC ground state occurs within ≈13 μs, while the MFPT associated with the formation of the S-bend structure is ≈17 μs. Intriguingly, the relaxation to the RC ground state from the different N* conformations of Aβ42 takes ≈260 ns, which is about 40 times slower than that of Aβ40. This remarkable difference in time scales is a consequence of the subtle variations in landscape frustration. Once formed, the fibril-like states of Aβ42 can survive longer and mediate further assembly processes.

### N* states are optimal templates for dimerization

**Fig. 5.**
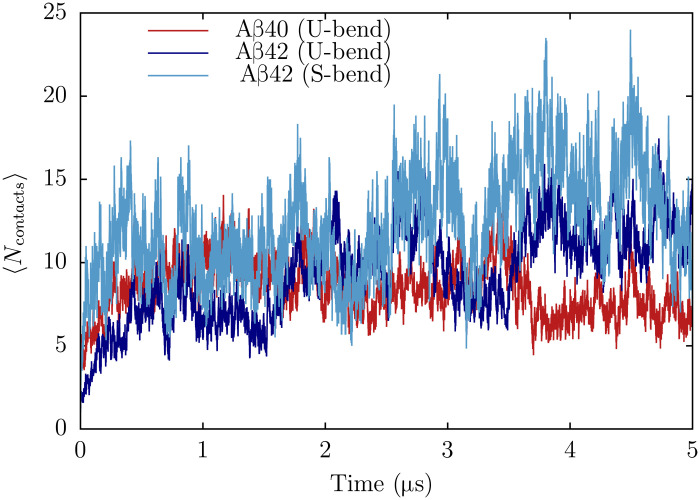
N* states readily form dimers. Dimerization kinetics for Aβ40 and Aβ42 when different N* states are allowed to coalesce. The extent of dimerization is monitored using 〈*N*_contacts_〉, the number of interchain contacts. Each dimerization profile is generated by averaging the time-dependent changes of *N*_contacts_ over 20 independent trajectories.

To ascertain the self-assembling propensities of different monomer conformations, we probed the dynamics of dimerization using the number of interchain contacts, 〈*N*_contacts_〉 as the order parameter. We assume that a dimer is formed when 〈*N*_contacts_〉 exceeds 5 (see the Supplementary Materials for details). As is evident from the relatively large values of 〈*N*_contacts_〉, dimerization is most effective for S-bend motifs of Aβ42 (Fig. 5). In contrast, the U-bend structures of Aβ42 appear less efficient at templating, with 〈*N*_contacts_〉 attaining large values only at longer time scales. Dimer formation is the least favored for Aβ40, with fewer interchain contacts forming along the trajectories. Hence, the extent of frustration on the energy landscape does seem to correlate with the propensity of dimerization.

#### Dimerization seeded from mixed states

We observe that dimerization could also occur if one of the monomers is in an N* state and the other is in a RC-like configuration. These “mixed” dimers formed by the U-bend structures of Aβ40 and Aβ42 are highly labile and periodically melt and reform along the trajectories (fig. S9). In contrast, mixed dimers formed by the S-bend and RC structures are relatively stable (fig. S9). Dimers exhibiting a large number of interchain contacts are also transiently formed through the assembly of U-bend and S-bend conformations of Aβ42 (fig. S9). Overall, it appears that the S-bend motif could act as an assembly template not only for N* states but also for RC-like configurations.

#### Dimerization seeded from RC states

When simulations were initiated from RC configurations, the number of interchain contacts, 〈*N*_contacts_〉 formed along the trajectories are generally lower (fig. S9). This suggests that RC conformations do not act as optimal templates for dimerization.

**Fig. 6.**
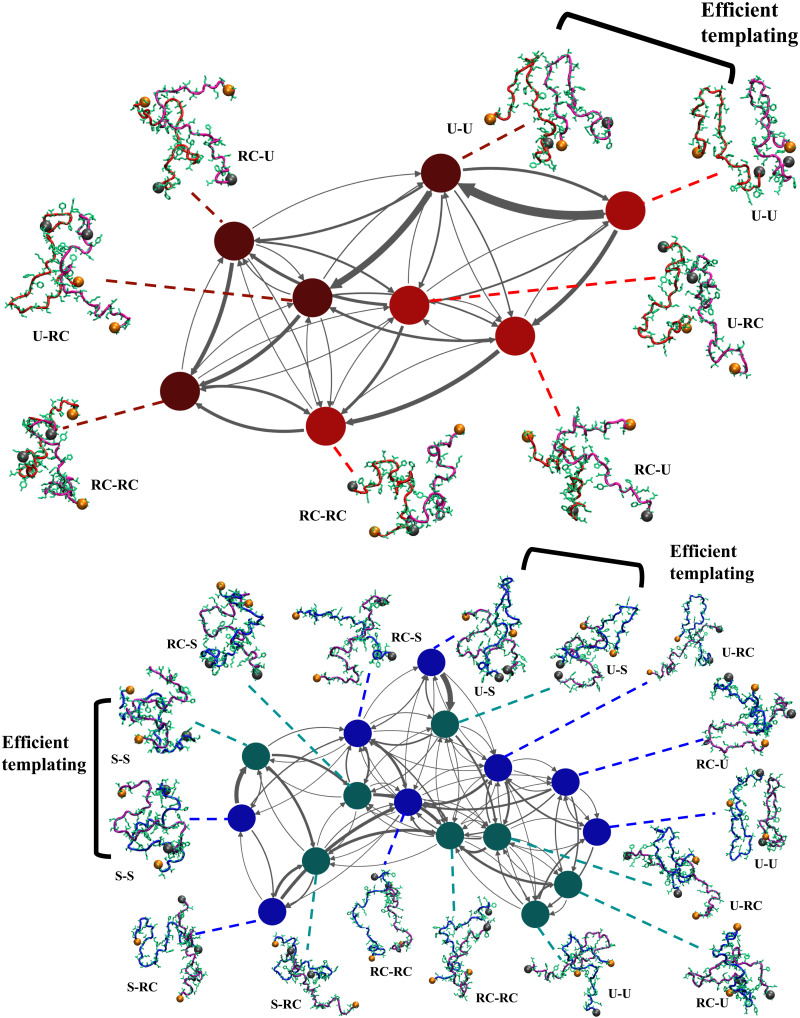
Different dimerization routes for Aβ40 and Aβ42. Transition networks illustrating the different dimerization routes for Aβ40 (top) and Aβ42 (bottom). The network was constructed by discretizing the trajectories and identifying the different substates using a HMM. The substates represent the nodes (denoted as circles) of the network. The thickness of the arrows connecting the different nodes is proportional to the transition probabilities. For Aβ40, the nodes denoting the monomers are shown in red, and those denoting the dimers are shown in maroon. For Aβ42, the monomers are represented by blue nodes, and the dimers are represented by teal nodes. Representative snapshots corresponding to each substate are also shown. The N termini are depicted as orange spheres, and the C termini are shown as gray spheres. The chains within each substate could either be in an N* (U-bend for Aβ40 and U-bend or S-bend for Aβ42) or an RC configuration. Efficient templating implies that the N* state serves as a template for inducing the RC → N* transition so that dimer formation and other downstream assembly occur readily.

To glean further insight into the dimerization pathways, we constructed transition networks connecting the different substates using hidden Markov models (HMMs) (*67*). The network for Aβ40 consists of eight states (shown as nodes in Fig. 6). The N* states serve as efficient templates and dimers in which both the chains have fibril-like signatures form readily. However, these structures relax to thermodynamically more stable configurations within the dimer basin, in which only one of the chains is fibril-like (U-bend-RC) or both the chains are disordered (RC-dimer). This suggests that the nucleus that will sustain growth exceeds two and is most likely close to six (*68*). The connectivity of the transition network suggests that the RC-dimer (lowest-energy structure within the dimer basin) is less likely to form through coalescence of two monomeric RC states. In other words, kinetically favored routes for dimerization involve a cascade of free energy excited states, and a direct transition between the monomer and dimer basins seems less efficient.

The network for Aβ42 comprises 16 states and has a more complex topology (Fig. 6). As is evident from the large transition probability (Fig. 6), the S-bend dimer can form through efficient templating of monomer structures. The mixed dimer unit, where one of the chains is in a U-bend configuration and the other is a S-bend, also assembles rapidly from isolated monomers. These dimers subsequently relax to the more stable RC-dimer through different routes involving multiple intermediates, including the S-bend-RC and the U-bend-RC states.

The connectivity of the network suggest that the U-bend dimer has a low probability of forming via direct coalescence, and once formed, it rapidly relaxes to other structures, including the U-bend-RC and the RC-dimer. This equilibration within the dimer basin reflects the growth in 〈*N*_contacts_〉 at long time scales, when the dimerization reaction is initiated from the U-bend structures of Aβ42 (Fig. 5).

Similar to Aβ40, the probability of a direct transition between the ground states of the monomer and the dimer is low. For dimerization to be efficient, the pathway must proceed through free energy excited states within the different basins, thus supporting the expectations based on the N* theory.

### Conformational transitions to fibril-like states and implications of Ostwald’s rule

We calculated the kinetics associated with the transition from the free energy ground state (RC-like structures) to N* states (with χ_fib_ ≥ χ*_c_*) using the distributions of first passage times (FPTs), *P*(τ_FPT_), where

$$P(\tau_{\mathrm{FPT}})=\frac{1}{N_{t}}\sum_{i=1}^{N_{t}}[\delta (1-\tau_{i})]$$ (4)

In Eq. 4, τ*_i_* is the time in the *i*-th trajectory when a structure with χ_fib_ ≥ χ*_c_* is visited for the first time, and *N_t_* denotes the total number of independent trajectories. We used the jackknife method (*69*) to determine the errors in the estimates of MFPTs.

**Fig. 7.**
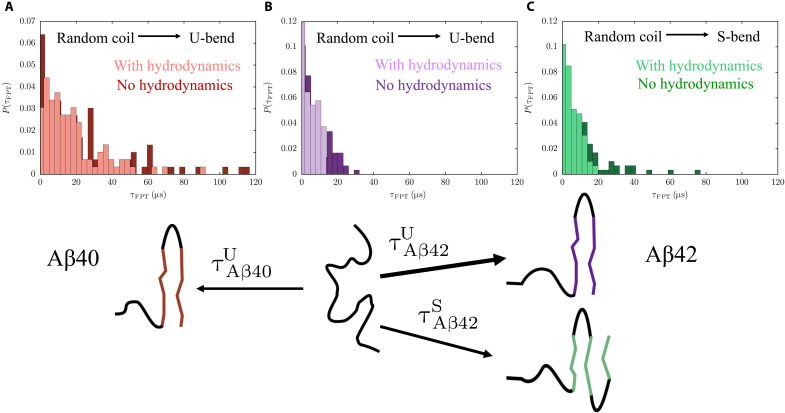
Distributions of FPTs for RC → N* transitions. Histograms depicting the distributions of FPTs corresponding to the transition from the disordered ground state to the fibril-like N* states. A schematic describing the different transitions is shown below the histograms. (**A**) FPT distribution for the transition from an equilibrium RC (free energy ground state) to the U-bend fibril-like structures in Aβ40. In the absence of hydrodynamic interactions, the MFPT,$\tau_{A\beta 40}^{U}$, for this transition is ≈25 μs. (**B**) FPT distribution for the transition from an equilibrium RC to the U-bend fibril-like conformations in Aβ42. In the absence of hydrodynamic interactions, the MFPT,$\tau_{A\beta 42}^{U}$, for this transition is ≈9 μs. (**C**) FPT distribution for the transition from RC-like structures to the S-bend fibril-like conformations. The MFPT $\tau_{A\beta 42}^{S}$ is larger than $\tau_{A\beta 42}^{U}$ and is around 13 μs. In all cases, inclusion of hydrodynamic interactions shortens the tails of the FPT distributions and accelerates the conformational transitions to the N* states.

**Fig. 8.**
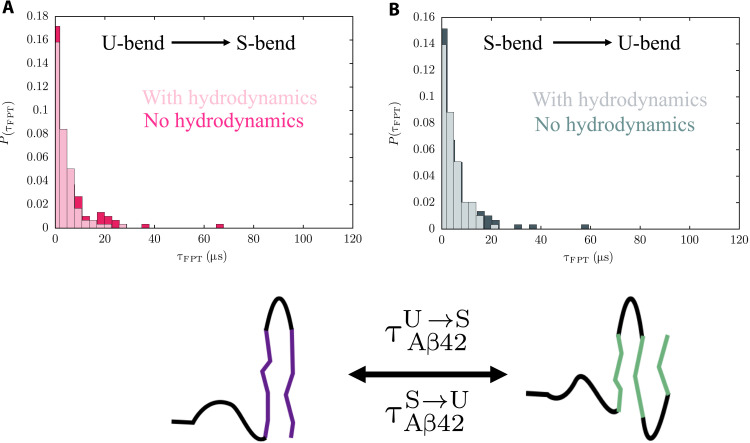
Distribution of FPTs for transitions between U-bend and S-bend conformations of Aβ42. The FPT distributions corresponding to the conformational switch between the U-bend and S-bend fibril-like conformations in the ensemble of Aβ42. (**A**) FPT distribution for the U-bend to S-bend transition. (**B**) FPT distribution for the S-bend to U-bend transition. In both the cases, the MFPT is ≈7 μs. The FPT distributions do not exhibit long tails when hydrodynamic interactions are taken into account. The MFPTs corresponding to the conformational switch also become smaller.

The distributions of the FPTs are shown in Figs. 7 and 8. The widths of *P*(τ_FPT_)s suggest that the transitions could occur through a multitude of pathways. All the distributions are approximately Poissonian, suggesting that the transitions to the N* states do not involve any long-lived intermediates, and the underlying kinetics can be appropriately described by a two-state model. To further assess the validity of the two-state kinetics, we analyzed the log(τ_FPT_) distributions, which can resolve signatures corresponding to multiple relaxation time scales (*70*). For both Aβ40 and Aβ42, the log(τ_FPT_) distributions are largely unimodal and can be fit using single Gaussian functions (figs. S10 and S11). These features are consistent with the overall topology of the TRDGs and imply that there are no deep kinetic traps on the energy landscape.

The transitions to fibril-like monomer configurations are considerably faster in Aβ42 as compared to Aβ40 (Fig. 7). The MFPT, $\tau_{A\beta 40}^{U}$, associated with the transition from an equilibrium RC-like conformation to a U-bend structure in Aβ40 is ≈25±1 μs. The corresponding MFPT for Aβ42, $\tau_{A\beta 42}^{U}$, is around three times smaller (≈9±0.4 μs). Although the S-bend structure is thermodynamically preferred (*21*), it forms on a longer time scale as compared to the U-bend conformation ($\tau_{A\beta 42}^{S}$ ≈ 13±0.5 μs). The MFPTs are in good agreement with those estimated from the transition networks using graph transformation, indicating that the TRDG construction preserves the key free energy barriers.

The ordering kinetics of polymorphic structures in Aβ42 is in accord with Ostwald’s rule of stages (*71*). The thermodynamically favored S-bend forms at a slower rate than the less stable U-bend topology. Ostwald’s rule was originally proposed in the context of crystal polymorphism in materials (graphite and diamond structures in carbon). However, it seems to be far-reaching and, as illustrated here, could encompass disorder-to-order transitions in IDPs.

The conformational switch between the U-bend and S-bend forms in Aβ42 is faster than the corresponding RC → N* transitions, with $\tau_{A\beta 42}^{U\to S}∼\tau_{A\beta 42}^{S\to U}\approx$ 7±0.5 μs (Fig. 8). This observation implies that for most of the conformational switching events, neither the U- or the S-bend structures have to access the RC state. Overall, the MFPTs for the different conformational transitions in Aβ peptides are in good agreement with the recent predictions from all-atom simulations (*72*).

#### Effect of hydrodynamic interactions

We find that the inclusion of hydrodynamic interactions not only accelerates the disorder to order (RC → N*) transitions, but also the conformational switch between the U-bend and S-bend motifs of Aβ42. The long tails of the FPT distributions, which arise due to an ensemble of non-optimal transition paths, become less pronounced in the presence of hydrodynamic interactions. Our observations are in accord with recent works that underscore the importance of hydrodynamic interactions in guiding the collapse transitions in proteins (*73*), and stepping of molecular motors on microtubule tracks (*74*).

For most transitions, there is a ≈1.3 to 1.5-fold decrease in MFPT in the presence of hydrodynamic interactions (table S4). However, hydrodynamic interactions have a more dominant impact on the RC → S-bend transition (Fig. 7C), with the MFPT being reduced by a factor of ≈2. We speculate that this enhancement of the reaction rate in the case of the S-bend motif could be linked to the more complex molecular mechanism (as compared to U-bend structures) underlying its formation.

#### Different definitions of N* states preserve the kinetic ordering of transitions

It is natural to ask if the relative time scales of the different transitions depend on the definition of the N* states? To test the validity of our key predictions, we extended our analysis to include additional experimental structures. When solid-state NMR structures (PDB ID: 2LMN and 2LMO) corresponding to twisted fibril morphologies (*75*) are used as references, the population of fibril-like U-bend conformations within the MCE is predicted to be even lower (table S5). The populations of non–U-type topologies, such as C-bend motifs (PDB ID: 6SHS) (*76*), and fully extended structures, which resemble the chains within the inner layers of a four-layered cross-β Aβ40 polymorph (PDB ID: 6W0O) (*77*), are also found to be negligible (table S5). As alternate references for Aβ42, we considered two additional S-bend fibril structures resolved using electron microscopy (PDB ID: 8AZT and 7Q4M) (*78*, *79*), an LS fibril structure resolved using cryo-EM (PDB ID: 5OQV) (*57*) and a tilde-shaped structure (PDB ID: 5AEF), which constitutes the dimeric core in a recently discovered Aβ42 polymorph (*80*). We find that the population of the S-bend structure and the time scale for its formation from the RC ground state are not sensitive to the choice of reference states (table S5), presumably because our model does not capture the differences in the side-chain packing among the different fibril models. The population of LS topologies is negligible within the MCE. The population of the tilde-shaped structures is approximately similar to that of the U-bend conformations, and the MFPT estimates suggest that they form earlier than the thermodynamically favored S-bend conformations. Hence, Ostwald’s rule of stages dictates the order of transitions to different N* states in Aβ42, even when additional fibril polymorphs are considered. We also find that irrespective of the choice of reference states, the RC → N* transition is slower for Aβ40 as compared to Aβ42.

## DISCUSSION

In this work, we used the SOP-IDP model (*21*) to characterize the structural and kinetic heterogeneity of Aβ40 and Aβ42 monomers. Structural heterogeneity, which is evident at the monomer level, has recently been illustrated to play an important role in the aggregation cascade in insightful single-molecule fluorescence imaging experiments (*7*). The topography of the free energy landscapes for both the sequences suggest that the RC-like ground state is readily accessible from the N*-like states found in the fibril polymorphs, over a wide range of experimental conditions, such as temperature, pH, or salt concentration. Hence, it is expected that the RC-like conformations belonging to the ground state determine the different thermodynamic observables, including residue-residue contact maps, secondary structure profiles, and residue-dependent chemical shifts.

**Fig. 9.**
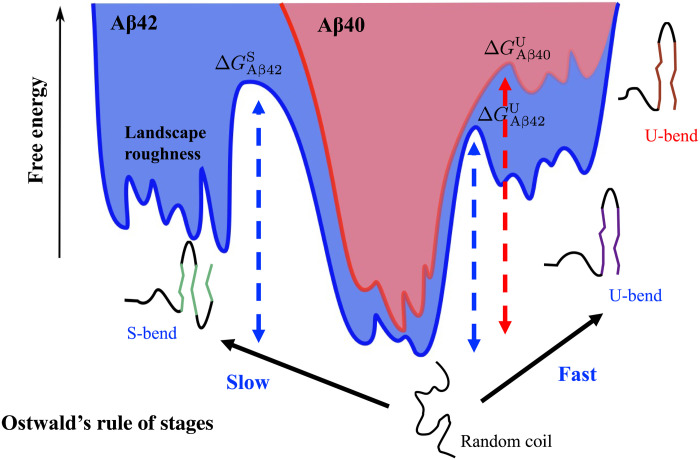
A model energy landscape illustrating Ostwald’s rule of stages. A schematic of the free energy landscape for Aβ40 (red) and Aβ42 (blue). The landscape roughness is enhanced in Aβ42 (especially near the S-bend motif) compared to Aβ40. This subtle variation in topography could have important implications on the self-assembling propensities of the monomers. The relative positions of the different N* states relative to the disordered ensemble (free energy ground state for both Aβ40 and Aβ42) are also shown. As is evident from the relatively high free energy barrier, $\text{Δ}G_{A\beta 40}^{U}$, the transition to the U-bend form is slower in the case of Aβ40. The transitions to the fibril-like conformations occur much faster in Aβ42 and are dictated by Ostwald’s rule of stages. The U-bend topology appears before the S-bend topology (i.e. $\text{Δ}G_{A\beta 42}^{U}<\text{Δ}G_{A\beta 42}^{S}$) because the later is thermodynamically favored.

The dominance of the featureless ensemble corresponding to the ground state might give the erroneous impression that not much can be discerned from the study of monomers. However, we find that monomer conformations, which have remnants of the fibril state, are encoded as excitations in the free energy landscape (Fig. 9). In other words, the “ordered” structures appear as high-lying free energy minima. Such a topography seems consistent with the recently proposed “inverted funnel” free energy landscape picture proposed for IDPs (*81*).

### Relaxation kinetics in the free energy landscape

Time-resolved spectroscopy has emerged as a potent tool for the characterization of the multi-tiered conformational dynamics in IDPs (*34*, *35*, *63*). nFCS showed that the reconfiguration time of Aβ40 and Aβ42 monomers is ≈35 ns (*34*), and neither peptide exhibits conformational dynamics on the microsecond to millisecond time scale. On the other hand, Trp-Cys contact quenching experiments were used to estimate an upper bound of around 1 μs for the intramolecular diffusion of Aβ peptides (*63*). In contrast to global chain relaxation, localized motions of the peptide backbone, including chain tumbling, and segmental dynamics occur on much faster time scales (≈10 ns or less) and have recently been reported using NMR spin-relaxation techniques (*35*).

The dynamics of Aβ monomers are hierarchically organized, with the relaxation times ranging from ≈3 to 100 ns. The slower time scales correspond to global chain relaxation, while the faster ones correspond to local deformation modes of the peptide chains. In accord with nFCS experiments (*34*), we do not find any relaxation process in the microsecond to millisecond regime.

### Organization of Aβ monomer landscapes differ from multifunctional IDPs

Some studies (*82*, *83*) speculate that certain IDP sequences may exhibit glass-like behavior and switch conformations over extremely long time scales. The free energy landscape of the p53 upregulated modulator of apoptosis (PUMA) peptide, for example, is highly frustrated (almost glass-like) in the absence of its binding partner and does not exhibit a well-defined global minimum (*84*). The multifunnel nature of the landscape encodes the multifunctional behavior of PUMA, by virtue of which the peptide can bind to its partner Mcl-1 in different poses. However, no such behavior for Aβ peptides is immediately apparent from the organization of the energy landscapes (Figs. 1 and 2) and relaxation time scales [Fig F1][Fig F2](Fig. 3). It is likely that the length of the IDP has to be sufficiently long enough or there must be stretches of sticky residues (e.g., polyglutamines) in the sequence to see a crossover to a multifunnel picture and, perhaps, glassy dynamics. A recent study has suggested such a scenario for a mutant huntington protein (*82*).

Previous studies (*85*, *86*) have characterized the energy landscapes of Aβ assemblies as multifunneled, corresponding to different arrangements of the monomer chains. It is likely that in the context of amyloid formers, multifunnel character does not have functional implications (such as for the PUMA peptide), but rather correspond to dysregulated states that are populated along the aggregation cascade.

### Landscape frustration peaks near N* states

Despite having globally similar topographies, the energy landscapes of Aβ40 and Aβ42 exhibit different extents of local frustration, particularly in the regions with high N* populations. We believe that this does have important implications in dictating the distinct aggregation propensities of Aβ40 and Aβ42.

The enhanced frustration impedes the reconfiguration in case of Aβ42, thus prolonging the lifetime of the N* states, which greatly facilitates self-assembly events. Because the local frustration decreases the reconfiguration by *e*^(δϵ/*k*_B_*T*)^2^^ (where δϵ/*k*_B_*T* is the roughness scale), even a small change in δϵ could have a large effect on reconfiguration (*87*)] and hence the lifetime of the N* state.

### Ostwald’s rule and structural polymorphism

The conformational transition from the disordered state to fibril-like structures in the monomers occurs on the microsecond time scale. The transition to the fibril-like monomer state is faster in Aβ42 compared to Aβ40. This observation implies that Aβ42 is better poised to form the early oligomers along the aggregation cascade (*30*).

Unexpectedly, we find that in Aβ42, the transitions to fibril-like structures are in accord with Ostwald’s rule of stages, which is often used in the solid-state community to predict the appearance of different crystal polymorphs. In other words, the U-bend fibril polymorph is likely to form first, while the S-bend polymorph, which is thermodynamically more stable, should appear on longer observation time scales. Of course, the precise order of events will be dictated by the underlying energy landscape, which can be tilted by changes in external conditions.

As shown here, Ostwald’s rule is manifested even during the early stages of the disorder to order transition in the MCE. However, it could also have important consequences during the late events of aggregation. A recent study has shown that Ostwald’s rule dictates the structural transitions in dipeptide supramolecular polymers (*71*). These findings could be important to advance our understanding of fibril polymorphism, particularly for sequences with low-complexity (LC) domains (*88*–*90*), where even subtle variations in experimental conditions or preparation protocols result in different fibril morphologies.

It is worth pointing out that Ostwald’s rule also explains tidily the ordering kinetics in the low-complexity domain FUS protein and various related constructs. In the FUS-LC sequence (residues 1 to 214), only residues 39 to 95 form S-bend type fibrils (core1) (*88*). On the other hand, residues 112 to 150 in a truncated variant of FUS-LC (residues 108 to 214) (*89*) form a fibril with a U-bend topology (core2), which is marginally destabilized with respect to core1. In an intriguing new development (*90*), it has been shown that another C-terminal variant of FUS-LC (residues 141 to 214) could also form fibrils (core3), with residues 155 to 190 forming the core region. Experiments suggest that core3 forms before core2, which, in turn, forms before core1. The stabilities of the three cores show exactly the opposite trend (*90*). Thus, there is an inverse relationship between thermodynamic stability and fibril formation rates, further affirming Ostwald’s rule of stages, that we have established in this study in the context of Aβ aggregation.

### N* theory for Huntington (htt) polyglutamine (Q)

The N* theory provides support to the kinetic scheme developed for dimer formation from the disordered ground state of the amphiphilic domain, Q_7_ (*91*). Using relaxation dispersion NMR measurements, it was shown that the ground state of Q_7_ is disordered (population ≈ 95%), just as in Aβ peptides. The productive route to Q_7_ dimerization occurs with small probability (≈2%) on a time scale of about 20 μs. The unstable dimers coalesce to form stable tetramers. The detailed quantitative kinetic analysis presented in the NMR study (*91*) is in accord with the N* theory elucidated here. In accord with the mechanism for Aβ dimer formation, we envision that the most productive path must involve Q_7_→ $Q_{7}^{*}$, which is followed by $Q_{7}^{*}+Q_{7}^{*}\to {\left(Q_{7}\right)}_{2}$. From this picture, it follows that $Q_{7}^{*}$ would be the sparsely populated (<2%) excited state in the monomer ensemble. The accumulating experimental evidence [see e.g. (*22*, *91*, *92*)] backed up by computations shows that the N* theory is a general mechanism in the initiation of protein aggregation. A corollary is that the characterization of excited states is necessary to assess the aggregation propensity of protein sequences.

Aβ40 and Aβ42 are the major isoforms implicated in AD, a progressive neurodegenerative disorder that affects a large fraction of the global population. Despite being present in relatively lower concentrations, Aβ42 aggregates nearly an order of magnitude faster than Aβ40 (*29*, *30*). For both the peptides, many experimentally relevant thermodynamic observables, such as *R*_g_ and fluorescence resonance energy transfer (FRET) efficiencies, are solely determined by the free energy ground state (consisting of RC-like structures). Hence, at the monomer level, a description based on ensemble averages alone is inadequate for rationalizing the apparent anomaly in the aggregation behavior of Aβ40 and Aβ42. In this study, we show that a detailed view of the free energy landscape (and not simply the thermodynamic ground state) is necessary for deciphering alloform-specific differences. The aggregation-prone, fibril-like monomer conformations (N* states) appear as excitations on the energy landscape and can be transiently accessed from the RC configurations on the microsecond time scale. The RC → N^*^ transition is several times faster for Aβ42, implying that it is kinetically predisposed to assemble compared to Aβ40. Unexpectedly, we find that for Aβ42, the least stable fibril-like structure (U-bend) forms faster than more stable ones (S-bend), in accord with Ostwald’s rule of stages (*71*), which was postulated nearly a century ago in the context of crystal polymorphism.

We show that the extent of landscape roughness, particularly in regions where assembly-prone conformations are most likely to be found, tidily explains the oligomerization propensity of Aβ40 and Aβ42. In particular, we find that the S-bend configurations, which are exclusively found in the conformational ensemble of Aβ42, act as very efficient templates for self-assembly. To probe the multitude of dimerization pathways, we constructed transition networks from our simulations using the formalism of HMMs (*67*). We find that kinetically favorable dimerization routes proceed through N* states, although the complex topology of the networks suggests that other possibilities could also exist. It is likely that dimerization and, by inference, higher-order oligomerization pathways are modulated by external conditions (such as pH, presence of crowders, or denaturants) (*93*) or by interactions with membranes (*94*).

## MATERIALS AND METHODS

### The SOP-IDP model

The simulations of the Aβ40 and Aβ42 peptides were carried out using the recently introduced SOP-IDP (*21*, *95*) model. In this model, each amino acid residue is represented using two interaction sites: a backbone bead (BB) centered on the *C*_α_ atom and a side-chain bead centered on the center of mass of the side chain (fig. S1). The SOP-IDP energy function is given by

$$U_{\mathrm{SOP}-\mathrm{IDP}}=U_{\mathrm{FENE}}+U_{\mathrm{EXV}}+U_{\mathrm{ELE}}+U_{\mathrm{BB}}+U_{\mathrm{BS}}+U_{\mathrm{SS}}$$ (5)

In Eq. 5, *U*_FENE_ describes the chain connectivity between the different interaction sites; *U*_EXV_ denotes purely repulsive interactions, which prevent any unphysical overlap between the beads; *U*_ELE_ describes the electrostatic interactions between charged amino acid side-chains. The final three terms, *U*_BB_, *U*_BS_, and *U*_SS_ are Lennard-Jones–type potentials, which describe the backbone-backbone (BB), backbone side-chain (BS), and side-chain–side-chain (SS), respectively. *U*_SS_ encodes the sequence specificity of the model and is based on the Betancourt-Thirumalai interaction map (*96*). The detailed functional form of the SOP-IDP potential and the force-field parameters are included in the Supplementary Materials.

### Monomer simulations

To probe the conformational dynamics of Aβ monomers, we carried out Brownian dynamics (BD) simulations in the high friction regime, corresponding to a solvent viscosity of 10^−3^ Pa.s. The inertial term in the Langevin equation can be ignored in this limit, and the motion of each bead *i* is described by

$${\dot{q}}_{i}=-\frac{1}{\gamma }\frac{\partial U}{\partial q_{i}}+{\text{Γ}}_{i}$$ (6)

In the overdamped limit, the natural unit of time $\tau_{\mathrm{HF}}=\frac{\gamma a^{2}}{k_{B}T}$. The typical values of the energy and length scales are 1 kcal/mol and 1 Å, respectively. Using these values, τ_HF_ is estimated to be 13.2 ps. The equations of motion were integrated using the Ermak-McCammon algorithm (*97*) using a time step of 0.05τ_HF_. To obtain meaningful estimates of FPTs and other kinetic observables, we carried 100 independent simulations for each Aβ monomer at 298 K. Each trajectory consisted of 2 × 10^7^ steps.

### Modeling hydrodynamic interactions

To simulate the effect of hydrodynamic interactions, we carried out BD simulations, where the motion of each bead is described by

$${\dot{q}}_{i}=-\sum_{j}\mu_{ij}\frac{\partial U}{\partial q_{i}}+{\text{Γ}}_{i}$$ (7)

In Eq. 7, μ*_ij_* denotes the conformation-dependent mobility tensor and is computed using a modified form of the Rotne-Pragar-Yamakawa approximation (*98*) introduced by Zuk *et al.* (*99*).

### Dimerization simulations

We probed the kinetics of dimerization in Aβ40 and Aβ42 using BD simulations. To mimic the critical protein concentration required for dimerization, we constrain the distance between the centers of masses of the two Aβ chains to the thermally averaged *R*_g_ of the monomer. The nonbonded interactions among beads in different chains were modeled as if they were part of the same monomer. In other words, if two residues *R*_1_ and *R*_2_ within the monomer have a collision diameter, σ_12_, and an interaction strength, ϵ_12_, then *R*_1_ and *R*_2_ in different Aβ chains would also interact with the same energy function.

The dimerization process was initiated from configurations where both Aβ chains adopted an N* (fibril-like) conformation (U-bend for Aβ40 and U-bend or S-bend for Aβ42), only one of the chains adopted an N* conformation, or both chains were in a RC configuration. For each initial condition, we carried out 30 independent simulations of 8 × 10^6^ steps, corresponding to ≈5.3 μs. The extent of dimerization was quantified using *N*_contacts_, the number of interchain contacts formed along the trajectory. An interchain contact was assumed to form if the distance between any two beads on different Aβ chains was ≤6 Å. Further details regarding the force-field, construction of the monomer free energy landscapes and transition networks for dimerization are included in the Supplementary Materials.

## Supplementary Materials

### This PDF file includes:

Supplementary Text Tables S1 to S5 Figs. S1 to S12 References

## Notes

View/request a protocol for this paper from *Bio-protocol*.
