# Supplementary material for: Energy landscapes of Aβ monomers are sculpted in accordance with Ostwald’s rule of stages
Source: Sci Adv. 2023 Mar 22;9(12):eadd6921. doi: 10.1126/sciadv.add6921 (PMC10032606; doi:10.1126/sciadv.add6921)
Supplement: Supplementary file 1 — Supplementary Text Tables S1 to S5 Figs. S1 to S12 References [file sciadv.add6921_sm.pdf]

Science Advances
AAAS

Supplementary Materials for

Energy landscapes of Aβ monomers are sculpted in accordance with Ostwald's rule of stages

Debayan Chakraborty *et al.*

Corresponding author: Dave Thirumalai, dave.thirumalai@gmail.com

Sci. Adv. **9**, eadd6921 (2023)
DOI: 10.1126/sciadv.add6921

This PDF file includes:

Supplementary Text
Tables S1 to S5
Figs. S1 to S12
References

The SOP-IDP model

The Self-Organized Polymer (SOP) model for intrinsically disordered proteins (abbreviated as SOP-IDP) was used to simulate the conformational dynamics of the Aβ40 and Aβ42 monomers. The SOP-IDP model quantitatively reproduces the scattering profiles of a diverse range of IDP sequences, of varying sequence composition, lengths, charge densities (*95*). In the SOP-IDP model, each amino acid residue is represented using two interaction sites: a backbone bead (BB) centered on the C_α atom, and a side-chain bead (SC) centered on the center-of-mass of the side-chain (Figure S1). The energy function for the SOP-IDP model is given by:

$$\begin{aligned} U \;=\; & -\sum_{i=1}^{N_B} \frac{k}{2} R_0^2 \log\left(1 - \frac{(r_i - r_{ref,i})^2}{R_0^2}\right) + \sum_{i=1}^{N_{loc}} \epsilon_{loc} \left(\frac{\sigma_i}{r_i}\right)^6 + {}' \sum_{i,j} \frac{e_i e_j \exp(-\kappa r_{ij})}{\varepsilon r_{ij}} \\ & + \sum_{i=1}^{N_{BB}} \epsilon_{BB} \left[\left(\frac{\sigma_i}{r_i}\right)^{12} - 2\left(\frac{\sigma_i}{r_i}\right)^6\right] + \sum_{i=1}^{N_{BS}} \epsilon_{BS} \left[\left(\frac{\sigma_i}{r_i}\right)^{12} - 2\left(\frac{\sigma_i}{r_i}\right)^6\right] \\ & + \sum_{i=1}^{N_{SS}} \epsilon_{SS} \left|\epsilon_i - 0.7\right| \left[\left(\frac{\sigma_i}{r_i}\right)^{12} - 2\left(\frac{\sigma_i}{r_i}\right)^6\right] \end{aligned} \quad (1)$$

The first term in Eq. 1 denotes the finitely extensible nonlinear elastic (FENE) potential, which accounts for the chain connectivity, with $r_{ref,i}$ representing the equilibrium distance between the bonded moieties. Purely repulsive excluded volume interactions are included (second term in Eq. 1) to prevent any unphysical overlap between the beads. The third term describes the Debye-Hückel potential, which accounts for the electrostatic interactions between the different charged residues. The final three terms in Eq. 1 describe the backbone-backbone (BB), backbone-sidechain (BS), and sidechain-sidechain (SS) interactions, respectively. The parameter ϵ_i corresponds to the Betancourt-Thirumalai matrix element (*96*), and encodes the sequence-specificity of the SOP-IDP model. There are three adjustable parameters in the model: ϵ_{BB}, ϵ_{BS}, and ϵ_{SS}. Their values were determined using a learning procedure described elsewhere (*95*). The values of the different parameters used in the SOP-IDP model are given in Tables S1 and S2.

Parameter	Value
R_0	2.0 Å
k	20.0 kcal/(mol Å^2)
ϵ_{loc}	1.0 kcal/mol
ϵ_{BB}	0.12 kcal/mol
ϵ_{BS}	0.24 kcal/mol
ϵ_{SS}	0.18 kcal/mol

Table S1: **Parameters of the SOP-IDP model.** Energy function parameters for the SOP-IDP model.

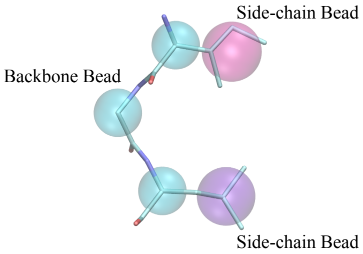

Figure S1. The SOP-IDP representation. A schematic illustrating the coarse-grained representation used in the SOP-IDP model, where each amino-acid is represented by two beads (interaction sites). One bead is centered on the backbone C_α atom, and the other one is centered on the center-of-mass of the side-chain. Here, the side-chain beads are shown in different colors to illustrate the sequence-specificity encoded in the SOP-IDP model. This figure is adapted from ref (*21*).

Residue	Radius (Å)	charge (e)
Gly	0.0	0.0
Ala	2.52	0.0
Val	2.93	0.0
Leu	3.09	0.0
Ile	3.09	0.0
Met	3.09	0.0
Phe	3.18	0.0
Pro	2.78	0.0
Ser	2.59	0.0
Thr	2.81	0.0
Asn	2.84	0.0
Gln	3.01	0.0
Tyr	3.23	0.0
Trp	3.39	0.0
Asp	2.79	-1.0
Glu	2.96	-1.0
His	3.04	0.0
Lys	3.18	1.0
Arg	3.28	1.0
Cys	2.74	0.0
backbone	1.90	0.0

Table S2: **Radii and charges for backbone and side-chain beads.** Parameters for the coarse-grained beads used in the SOP-IDP model.

Minimum	Free Energy (kcal/mol)	Error (kcal/mol)
Snapshot a	-6.30	-
Snapshot b	-3.09	0.09
Snapshot c	-2.85	0.19
Snapshot d	-3.26	0.08
Snapshot e	-2.90	0.20

Table S3: **Free energies of low-lying minima.** The free energies of different low-lying minima (depicted in the Transition Disconnectivity Graph (TRDG) (Figure 2)) for Aβ42. All errors are relative to the global minimum (snapshot a), and are estimated from the TRDG following the strategy of Krivov and Karplus (*37*).

Transition	τ_{HD} (μs)	τ_{NHD} (μs)
RC $\rightarrow$ U-bend (Aβ40)	19 $\pm$ 1.7	25 $\pm$ 1.0
RC $\rightarrow$ U-bend (Aβ42)	6 $\pm$ 0.4	9 $\pm$ 0.4
RC $\rightarrow$ S-bend (Aβ42)	7 $\pm$ 0.5	13 $\pm$ 0.5
U-bend $\rightarrow$ S-bend (Aβ42)	5 $\pm$ 0.5	7 $\pm$ 0.5
S-bend $\rightarrow$ U-bend (Aβ42)	5 $\pm$ 0.4	7 $\pm$ 0.6

Table S4: **Mean first passage times (MFPTs) for various transitions.** A comparison of the mean first passage times (MFPTs) for the various transitions obtained with hydrodynamic interactions (τ_{HD}) and without hydrodynamic interactions (τ_{NHD}). The errors in the MFPTs are estimated using the jack-knife method (*69*).

Reference structure (PDB ID)	Topology	Population (in %)	MFPT (μs)
2M4J	U-bend (Aβ40)	0.46 $\pm$ 0.02	25 $\pm$ 1.0
2LMN	U-bend (Aβ40)	0.10 $\pm$ 0.005	49 $\pm$ 2.7
2LMO	U-bend (Aβ40)	0.04 $\pm$ 0.003	60 $\pm$ 2.6
6SHS	C-motif (Aβ40)	0.05 $\pm$ 0.002	55 $\pm$ 3.5
6W0O	extended (Aβ40)	0.05 $\pm$ 0.002	48 $\pm$ 2.8
2BEG	U-bend (Aβ42)	1.45 $\pm$ 0.01	9 $\pm$ 0.4
2NAO	S-bend (Aβ42)	2.09 $\pm$ 0.04	13 $\pm$ 0.5
7Q4M	S-bend (Aβ42)	2.05 $\pm$ 0.04	11 $\pm$ 0.5
8AZT	S-bend (Aβ42)	2.17 $\pm$ 0.04	14 $\pm$ 0.8
5OQV	LS-motif (Aβ42)	0.08 $\pm$ 0.004	56 $\pm$ 1.8
5AEF	Tilde-shape (Aβ42)	1.4 $\pm$ 0.05	8 $\pm$ 0.6

Table S5: **N^* populations and MFPTs for different reference states.** Populations of N^* states, and MFPTs for RC $\rightarrow$ N^* transitions, when different experimental structures are used as references. The populations are estimated from BD trajectories generated from hundred different initial conditions. The total number of snapshots is 10^7. Hence, a population of 0.05% corresponds to $\approx$ 5000 conformations. The errors are estimated using the jack-knife method (*69*).

Identifying free energy local minima

The conformations sampled by the Brownian Dynamics (BD) trajectories were partitioned into discrete microstates using structural clustering. First, each conformation was mapped into its equivalent distribution of reciprocal interatomic distance (DRID) metric (*36*). As shown by Zhou and Calfisch (*36*), DRID clustering usually preserves the kinetic distances between any two given conformations, and is hence better suited for kinetic analysis as compared to other conventional structure-based metrics. In order to compute the DRID, two sets of atoms are required: the first is a set of m centroids, and the other is a list of atoms, N_{atom}. Each centroid, i, is associated with the three moments of the distribution of reciprocal distances (μ_i, ν_i, ζ_i), which describe the structural features of a given conformation. Hence, each conformation is described by a DRID vector of dimension $3m$. The distance, s_{jk}, between any two conformations j and k in the space of DRID vectors is given as:

$$s_{jk} = \frac{1}{3m} \sum_{i=1}^{m} \left[(\mu_i^j - \mu_i^k)^2 + (\nu_i^j - \nu_i^k)^2 + (\zeta_i^j - \zeta_i^k)^2\right]^{1/2} \tag{2}$$

The moments of distribution of the reciprocal distances are expressed as:

$$\mu_i = \frac{1}{N_{atom} - 1 - mb_i} \sum_i \frac{1}{d_{ij}} \tag{3}$$

$$\nu_i = \left[\frac{1}{N_{atom} - 1 - mb_i} \sum_i \frac{1}{(d_{ij} - \mu_i)^2}\right]^{1/2} \tag{4}$$

$$\zeta_i = \left[\frac{1}{N_{atom} - 1 - mb_i} \sum_i \frac{1}{(d_{ij} - \mu_i)^3}\right]^{1/3} \tag{5}$$

In the equations above, d_{ij} is the distance of atom j from centroid i; N_{atom} is the number of atoms; nb_i is the number of atoms bonded to centroid i. The summations in Eqs. 3-5 do not include the centroid i and the atoms covalently linked to it.

Subsequent to mapping in the DRID space, the conformational ensemble was clustered using a regular space clustering algorithm implemented within the *PyEMMA2.5* distribution (*100*). To identify distinct microstates, a cutoff of 0.15 was used for s_{jk}. Using this procedure, we identified 5956 microstates for Aβ40, and 6639 microstates for Aβ42. Each microstate i can be considered as a local minimum on the free energy landscape.

The free energy of minimum i was estimated as $F_i = -k_B T \ln(Z_i)$, where Z_i is the partition function. Here, Z_i is simply the number of conformations, N_i in cluster i (*37*). The full partition function, Z, is equal to the total number of conformations, N, sampled along the BD trajectories.

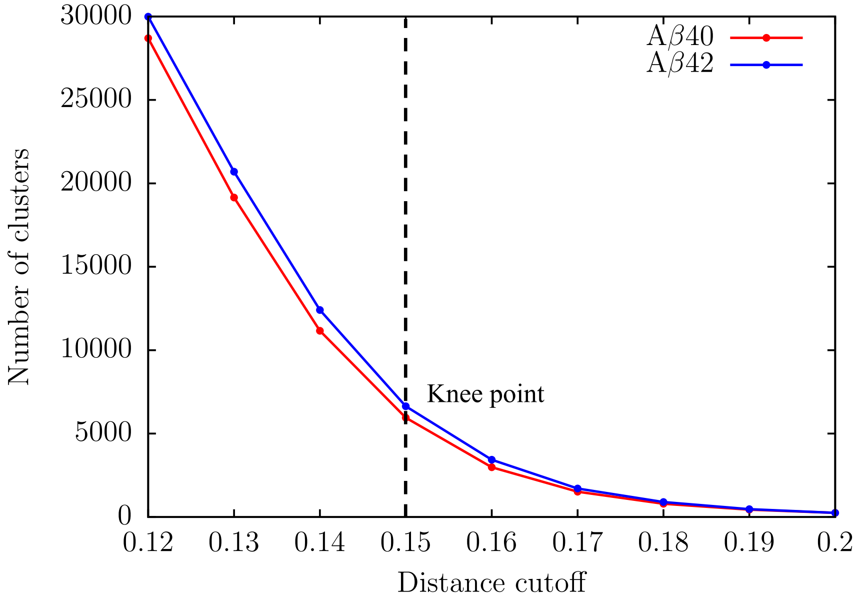

Figure S2. Determining the optimal number of clusters. Variation of the number of clusters with distance cutoff for Aβ40 (red) and Aβ42 (blue). The optimal distance cutoff in each case was determined from the knee point (denoted as a dashed line).

Calculation of effective barrier heights

The unimolecular rate constant for a transition between minima i and j can be approximately computed as (*37, 101*):

$$k_{ij} \approx \frac{k_B T}{h} \exp((-F_{ij} - F)/k_B T) = \left(\frac{k_B T}{h}\right) \frac{Z_{ij}}{Z} \tag{6}$$

In Eq. (6), for simplicity we approximate the attempt frequency, $\nu_0 \approx \frac{k_B T}{h}$. The effective free energy barrier along the pathway connecting minima i and j is F_{ij}, and F is the total free energy of the system; Z_{ij} denotes the partition function corresponding to the free energy barrier connecting minima i and j, and Z denotes the full partition function. The number of transitions, n_{ij}, between cluster i and j (or equivalently, the free energy local minima), can be directly computed from the BD trajectories. These counts were converted to symmetrized edge capacities, $c_{ij} = (n_{ij} + n_{ji})/2$, following Krivov and Karplus (*102*). The edge-capacities reflect the underlying dynamics of the system. Based on the isomorphism between the free energy landscape, and a flow-network consisting of capacitated edges, calculating the effective free energy barrier between two minima is equivalent to determining the maximum flow in the network (*37*). According to the Ford and Fulkerson theorem (*38*), the maximum flow (i.e. effective free energy barrier) between two nodes (or local minima) can be obtained from the number of “minimum-cuts”. Instead of finding the number of minimum cuts, k, for each pair of minima separately, we use the Gomory-Hu procedure (*103*) as implemented within the *networkx*

module. The Gomory-Hu procedure converts the flow-network with capacitated edges into a tree (i.e. between every pair of nodes, there is only connecting pathway), while preserving the minimum cuts between all pairs of nodes. Hence, only $k-1$ cuts need to computed.
The dynamics of the system can be described by the master equation (*101*):

$$\frac{d\mathbf{p}(t)}{dt} = \mathbf{W}\mathbf{p}(t), \tag{7}$$

where $\mathbf{p}$ represents a vector denoting the probabilities for the system to be in a particular minimum at time t, and $\mathbf{W}$ is the rate matrix. From the BD trajectories, the transition probability matrix, $\mathbf{T}(\tau)$ (where τ is the lagtime), is directly computable. It is related to the rate matrix as: $\mathbf{T}(\tau) = \exp(\mathbf{W}\tau)$. Hence, $\mathbf{W} = \ln(\mathbf{T}(\tau))/\tau$ (*37, 101*). If only the linear term in $\ln(\mathbf{T}(\tau))$ is considered then $\mathbf{W} = (\mathbf{T} - \mathbf{I})/\tau$. In other words, the matrix element, $w_{ij} = ((N_{ij}/N_i) - \delta_{ij})/\tau$, where N_{ij} is the number of minimum-cuts between i and j obtained using the Gomory-Hu algorithm (*103*).

From w_{ij}, the rate constant for the transition between minima i and j can be computed as: $k_{ij} = w_{ij}p_j = (N_{ij}/N)/\tau$. Comparing with Eq. (6), and taking into account that the total partition function, Z, is equal to the total number of conformations, N, sampled along the trajectories, the partition function , Z_{ij}, corresponding to the free energy barrier between minima i and j can be expressed as (*37*):

$$Z_{ij} = N_{ij}\frac{1}{\tau\nu_0} \tag{8}$$

The free energy barrier between minima i and j is given by:

$$F_{ij} = -k_BT\ln(Z_{ij}). \tag{9}$$

The free energy minima and the intervening barriers describe the connectivity of the landscape, and it is often convenient to consider them as kinetic transition networks (*41, 65*) from which thermodynamic or dynamical observables of interest can be extracted.

From the transition networks of Aβ40 and Aβ42, we estimated the rate constants (mean first passage times) corresponding to the transitions between the random-coil (RC) ground state and the different free energy excited states using a graph transformation procedure (*66*) implemented within the PATHSAMPLE code (*104*).

Visualizing the free energy landscapes

The energy landscapes were visualized in the form of disconnectivity graphs (*37, 39, 102*). In this representation, the landscape is partitioned into disjoint basins of attraction, by choosing an appropriate energy scale (Figure S3). The local minima within each basin are mutually accessible, whereas minima in different basins are connected by larger free energy barriers. A vertical line is drawn at each minimum (in this case, A-D), beginning at energy of that state. At a threshold of $E + \Delta E$, minima A and B (and also C and D) are connected together since the effective barriers

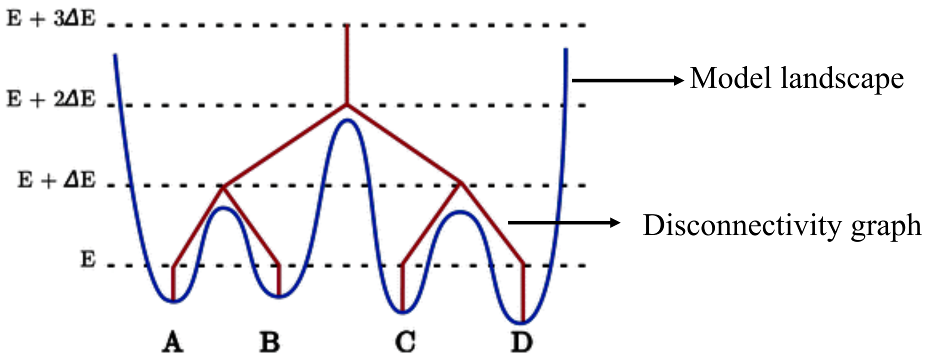

Figure S3. Construction of disconnectivity graphs. The disconnectivity graph representation (red) of a model free energy landscape (blue). Each local minimum is denoted by a vertical line starting at the location of the energy minimum. At any given energy threshold, $E + \Delta E$, minima that have an effective free energy barrier below the threshold are grouped into disjoint sets. The figure is adapted from ref (*105*).

separating these states are below the threshold. All the minima are connected at level $E + \Delta E$ because the barrier separating the two sets (A and B from C and D) lies below the threshold. In a sense, the disconnectivity graph representation captures the separation of time-scales inherent in the dynamics of the system. In contrast to other schemes, which rely on low-dimensional projections of the energy landscape onto predefined order parameters, disconnectivity graphs preserve the kinetic information (*102*).

Both transition disconnectivity graphs (TRDGs) as well as free energy disconnectivity graphs (FEDGs) are nearly equivalent ways of representing the energy landscape (Figure S5 and S6) (*37*). The TRDG depicts the complex dynamics on the landscape directly, in terms of transitions between minima. In this representation, there are no approximations in the description of the underlying dynamics, and the flow between the different minima is described in terms of the number of minimum cuts, N_{ij}. The effective barrier separating minima i and j is given by $-k_B T \ln(N_{ij})$. In a FEDG depiction, the free energy barriers are related to the unimolecular rate constants, and can be computed using Eqs (8) and (9).

The vertical lines in the TRDGs (Figures 1 and 2) and FEDGs (Figures S5 and S6) correspond to distinct free energy minima or clusters that were identified from the BD trajectories. The lines terminate at the energies corresponding to the minima. Within each cluster, conformations are structurally similar, with the distance, s_{jk} (see Eq. (2)) between the clusters being less than the chosen cutoff. For Aβ40 there are 5956 minima, and for Aβ42 there are 6639 minima. In both TRDGs and FEDGs, the horizontal axis is arbitrary, but in many cases, aligning the central vertical branch of the graph to the global minimum, provides a convenient visualization. For a particular minimum i connected to the central branch by a horizontal line at energy E, there exists a pathway to the global minimum (RC state) via a transition state lying below E. To

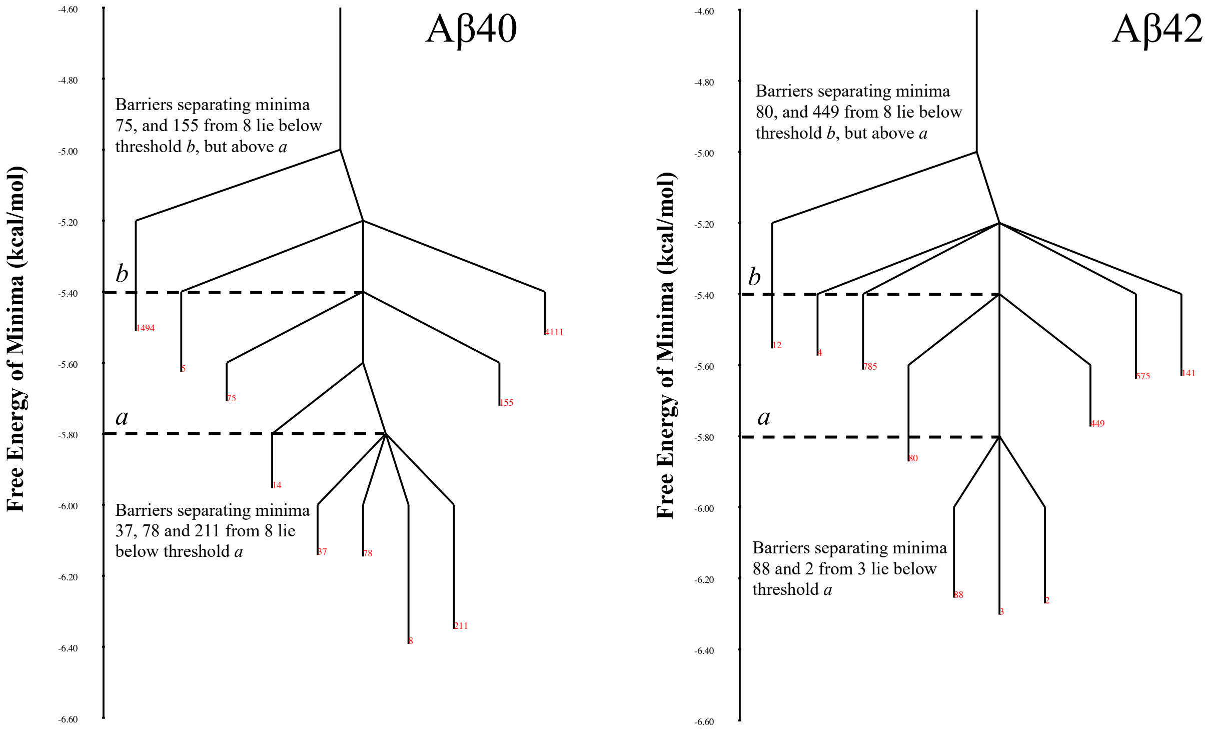

Figure S4. Coarse-grained Transition Disconnectivity Graphs (TRDGs) for Aβ40 and Aβ42. A coarse-grained version of the TRDG (showing only the connectivity corresponding to the 10 lowest minima) for Aβ40 (left) and Aβ42 (right). Here the flows between the minima, described in terms of the number of minimum-cuts, are shown directly (*18, 42*). Any minima that are separated from the global minimum by barriers lying below a chosen threshold (shown as dashed lines), are joined to the main branch at that threshold.

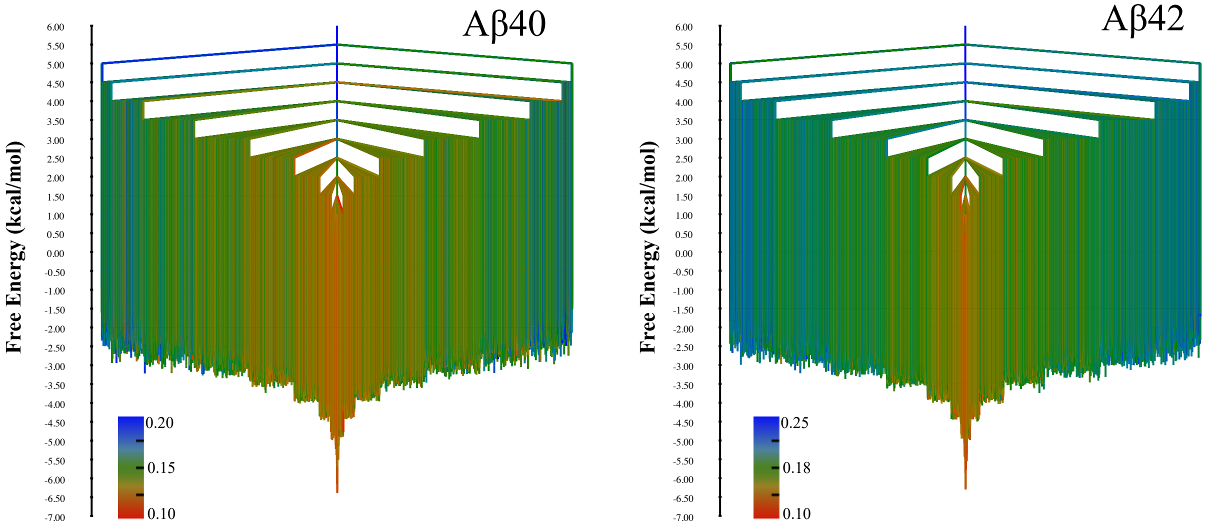

Figure S5. Free Energy Disconnectivity Graphs (FEDGs). Free energy disconnectivity graphs (FEDGs) for Aβ40 (left) and Aβ42 (right). The branches are colored according the scheme described in Figures 1 and 2. Unlike the TRDGs where the effective barriers are illustrated in terms of the flow between minima (number of minimum-cuts), the free energy barriers are explicitly computed using Eqs. (8) and (9). The FEDGs capture the overall flatness of the energy landscapes, a characteristic feature of many IDPs (*28, 43*).

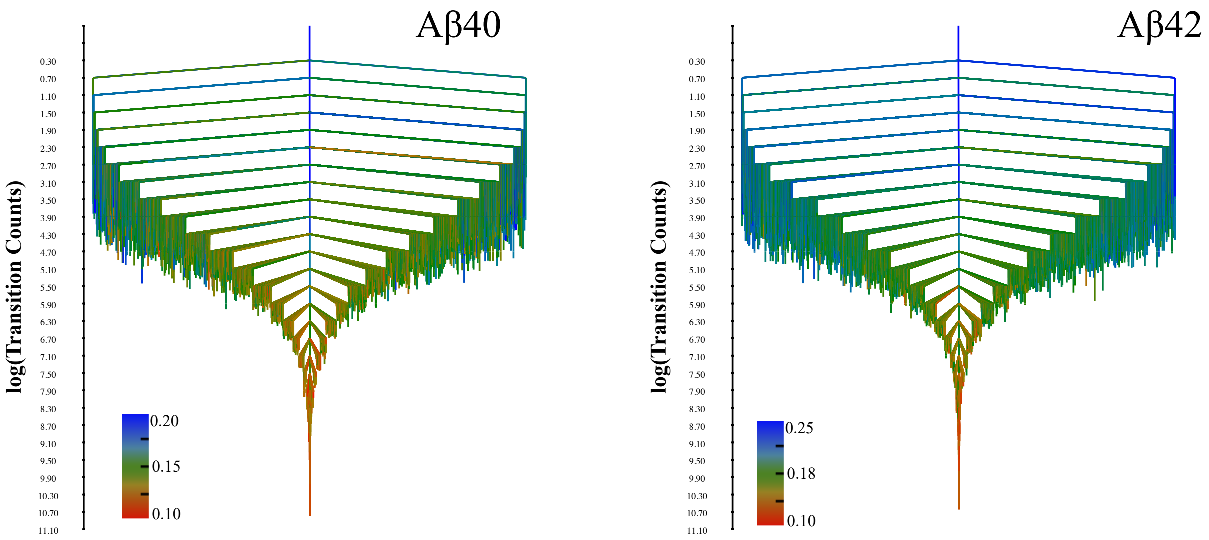

Figure S6. TRDGs depicting transition counts. Transition disconnectivity graphs (TRDGs) for Aβ40 (left) and Aβ42 (right). The branches are colored according to the scheme described in Figures 1 and 2. Unlike the TRDGs presented in the main manuscript, the y-axis denotes logarithm of the transition counts without thermal weighting.

illustrate this concept in the case of Aβ40 and Aβ42, a coarse-grained version of the TRDGs (showing only 10 minima) are displayed in Figure S4. The TRDGs and FEDGs were constructed using the disconnectionDPS code (*106*).

The errors in the free energies relative to the global minimum can be estimated from the count matrices used to construct TRDGs following the strategy of Krivov and Karplus (*37*). For minimum i, the statistical error in its free energy estimate is given by $\delta F_i = k_B T(2/N_{ij})^{1/2}$ where N_{ij} represents the number of transitions between minima i and j.

Estimation of landscape roughness density

The local frustration of the energy landscapes was quantified in terms of roughness density, ρ_{LB}. It is defined as the number of minima in the TRDG that branch off at a particular energy level (*61*).

$$\rho_{LB} = n_{branch}/\Delta G \tag{10}$$

In Eq. 10, n_{branch} is the percentage of free energy minima that branch off at a particular energy level, and ΔG represents the threshold used in the superbasin analysis.

Quantifying the degree of frustration on the energy landscape

The complexity of the energy landscape can be quantified in terms of a frustration metric, $f(T)$, introduced by Wales and coworkers (*64*). This metric describes how efficiently a system relaxes to the global minimum, and has been used to characterize the contrasting dynamics of structure-seekers, glass formers, as well as biomolecules (*64, 107, 108*). From the connectivity of the landscape, described in terms of minima and intervening barriers, $f(T)$ can be computed as a function of temperature.

$$f(T) = \sum_{i \neq GM} \frac{p_i^{eq}(T)}{1 - p_{GM}^{eq}(T)} \frac{F_i^{\dagger} - F_{GM}}{F_i - F_{GM}}. \tag{11}$$

In Eq. 11, $p_i^{eq}(T)$, is the equilibrium occupation probability of minimum i at temperature T, $p_{GM}^{eq}(T)$, denotes the occupation probability of the global minimum at temperature T, F_i is the free energy of minimum i, F_{GM} is the free energy of the global minimum, and $F_i^{\dagger}$ denotes the highest barrier height along the lowest energy path connecting minimum i to the global minimum. The frustration metric, $f(T)$, was computed for Aβ40 and Aβ42 as a function of temperature using the PATHSAMPLE code (*104*).

Definition of N* states

Aggregation-prone conformations (putative N* states) were identified from the conformational ensembles based on structural overlap with the monomer unit of the experimentally determined

Aβ40 and Aβ42 fibril structures. We computed $\chi_{fib}(t)$ using:

$$\chi_{fib}(t) = \frac{1}{N_{pairs}} \sum_{a,b}^{N_{pairs}} H(d - |r^i_{a,b}(t) - r^0_{a,b}|) \tag{12}$$

where $r^0_{a,b}$ is the distance between sites a and b in the reference structure. Conformations which exhibit $\chi_{fib}(t) \geq \chi_c$ are deemed to be aggregation-prone, and are collectively defined as belonging to the N* state. For Aβ40, we chose the monomer unit from the solid-state fibril structure (PDB ID: 2M4J) reported by Tycko and coworkers (*32*) as the reference state. To identify aggregation-prone conformations in the Aβ42 ensemble, we used the monomer units from two topologically different fibril structures reported by Riek and coworkers using solution-NMR as references, the U-bend fibril structure (*55*), and a S-bend fibril structure (*33*).

Constructing Transition Networks for Dimerization

Each snapshot in the dimerization trajectories was assigned a binary code (with values being either 0 or 1) based on the number of inter-chain contacts, $N_{contacts}$, and the structural overlap, $\chi_{fib}(t)$, with the monomer unit of the experimentally determined fibril structures. For Aβ40, the snapshots were indexed as (S, C_1^U, C_2^U), with S=1 denoting a dimer ($N_{contacts} \geq$ 5) and S=0 denoting isolated monomers. The label C_m^U=1 if the chain, C_m, (m is either 1 or 2) is fibril-like ($\chi_{fib}(t) \geq$ 0.30) and C_m^U=0 if the chain adopts a RC-like configuration.

For Aβ42, each configuration has two additional labels due to the possibility of the S-bend configuration. The snapshots in the trajectories were indexed as $(S, C_1^U, C_2^U, C_1^S, C_2^S)$. In this case, S=1 represents a dimer, and S=0 denotes isolated monomers. The label C_m^U=1 if the chain, C_m, (m is either 1 or 2) adopts a U-bend configuration ($\chi_{fib}(t) \geq$ 0.30) and C_m^U=0 if the chain is RC-like. Similarly, C_m^S=1 if the chain, C_m, adopts a S-bend configuration, and C_m^S=0 if the chain is in a RC-like configuration.

After assigning a discrete label to each snapshot, transition networks describing the dimerization process for Aβ40 and Aβ42 were constructed using the hidden Markov Model (HMM) formalism (*67*) implemented within the *PyEMMA2.5* distribution (*100*). The HMMs were estimated at a series of lag-times, and to construct the transition networks we chose a lag-time where the implied time-scales appear converged (Figure S12).

Generating all-atom structures from coarse-grained snapshots

The all-atom reconstruction of the coarse-grained (CG) structures was carried out in three stages. (1) First the BBQ code (*109*) was used to generate the all-atom representation of the backbone starting from the trace of the C_α positions available from the CG structure. (2) Following the backbone reconstruction, the side-chains for each CG conformation was generated using the SCWRL4 formalism (*110*). (3) Finally, the geometry of the reconstructed all-atom structure was further optimized to remove any unwanted steric clashes using the AMBERff12SB force field, in

conjunction with the Generalized Born solvent model parametrized by Onufriev, Bashford and Case (GB-OBC) (*111*). During the geometry optimization, positional restraints were applied to the C_α atoms to prevent any substantial distortion from the corresponding CG topology. The optimizations were carried out using the *sander* module available within the AMBER12 distribution (*112*).

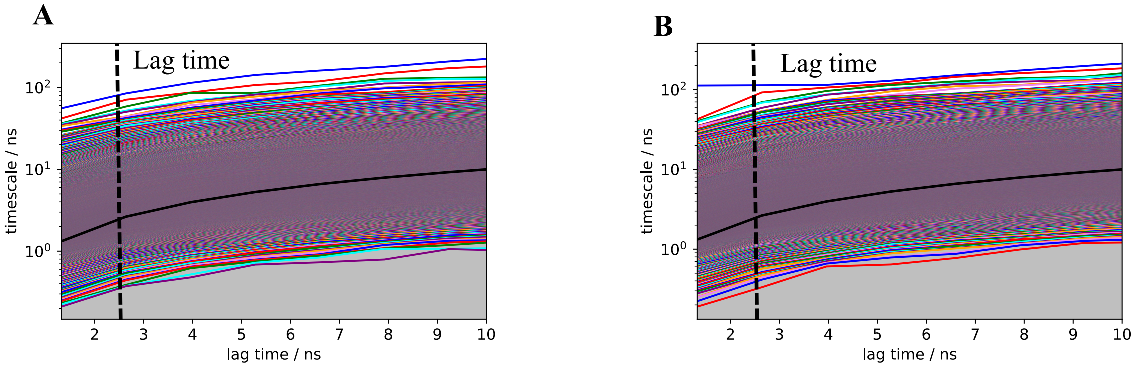

Figure S7. Determining lag-times. The implied time-scales for Aβ40 (A) and Aβ42 (B) are independent of the lagtime. To compute the relaxation time-scales within the Aβ40 and Aβ42 monomer ensembles, we chose a lagtime of 2.7 ns.

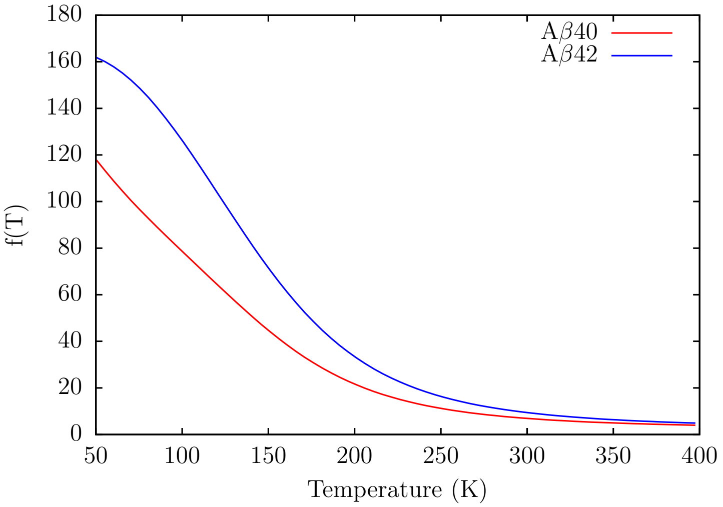

Figure S8. Frustration index as a function of temperature. The frustration index, $f(T)$, computed as a function of temperature from the transition networks of Aβ40 and Aβ42. As is evident, the $f(T)$ for Aβ42 is higher than that of Aβ40, particularly at low temperatures.

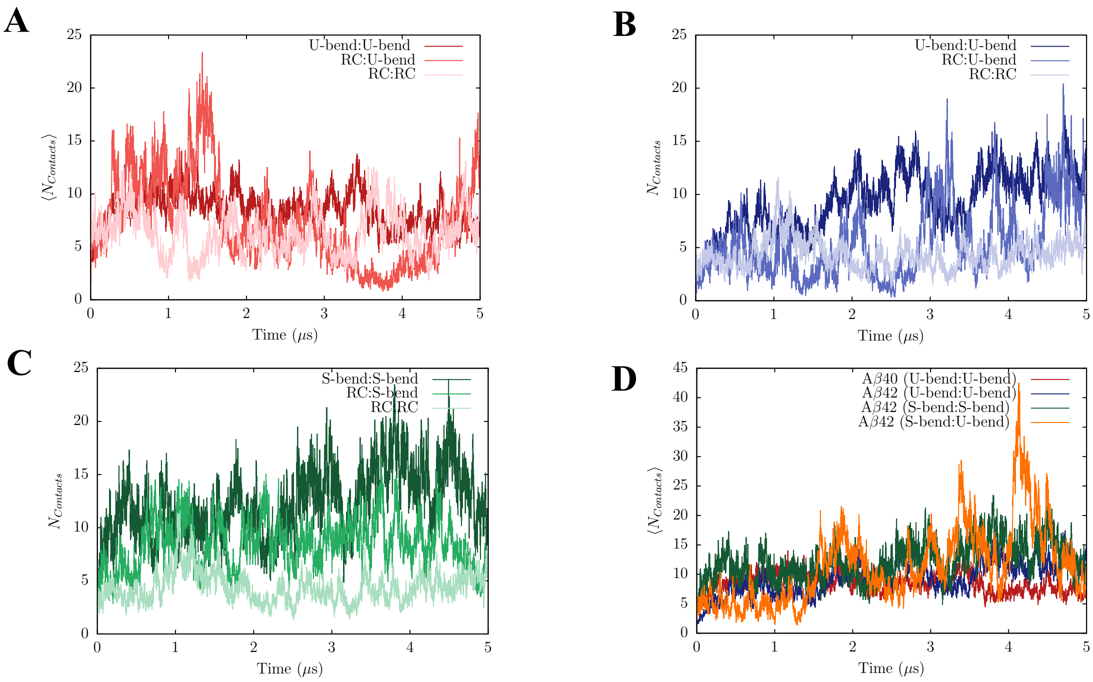

Figure S9. Dimerization seeded from different initial conditions. (A) Dimerization profiles of Aβ40 initiated from different configurations. A dimer is formed when the number of interchain contacts, $\langle N_{contacts} \rangle$ exceeds 5. When dimerization reactions are initiated from two RC configurations, there is little or no dimer formation. (B) Just as in Aβ40, dimers formed by a combination of a U-bend structure, and RC configuration, tend to be more dynamic (large fluctuations in $\langle N_{contacts} \rangle$. The propensity to form dimers is rather low, when dimerization reactions are initiated from two RC configurations. (C) The S-bend motif acts as an optimal template for dimer formation (high values of $\langle N_{contacts} \rangle$ throughout the simulation time). Dimers formed by a combination of S-bend and RC-like configurations are also thermodynamically stable compared to those formed by U-bend counterpart. The relatively low values of $\langle N_{contacts} \rangle$ suggest that the tendency to form dimers is rather low when the dimerization is initiated from two RC configurations. (D) Mixed dimers formed by a U-bend and a S-bend motif of Aβ42 are highly dynamic, and can transiently form a large number of interchain contacts.

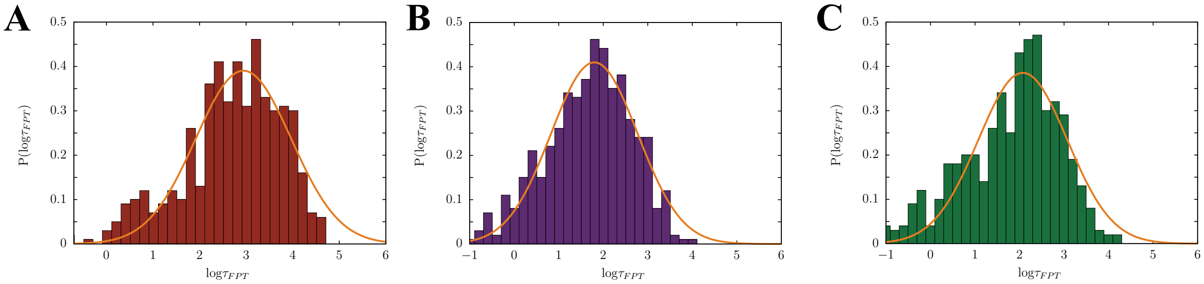

Figure S10. Distributions of log(τ_{FPT}) for RC→N* transitions. The distributions of log(τ_{FPT}) for (A) RC → U-bend transition in Aβ40; (B) RC → U-bend transition in Aβ42. (C) RC → S-bend transition in Aβ42. To minimize statistical noise, the distributions were generated using a larger sample-size (≈ 400 trajectories). In all cases, the distributions can be fit using single Gaussian functions (shown as solid yellow curves).

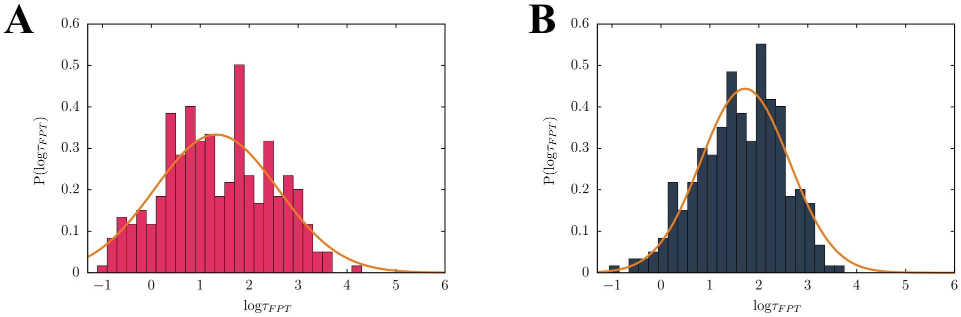

Figure S11. Distributions of log(τ_{FPT}) for transitions between U-bend and S-bend conformations in Aβ42. The distributions of log(τ_{FPT}) generated using a sample-size of ≈ 400 trajectories for (A) U-bend → S-bend transition in Aβ42; (B) S-bend → U-bend transition in Aβ42. Both the distributions can be fit using single Gaussian functions (shown as solid yellow curves).

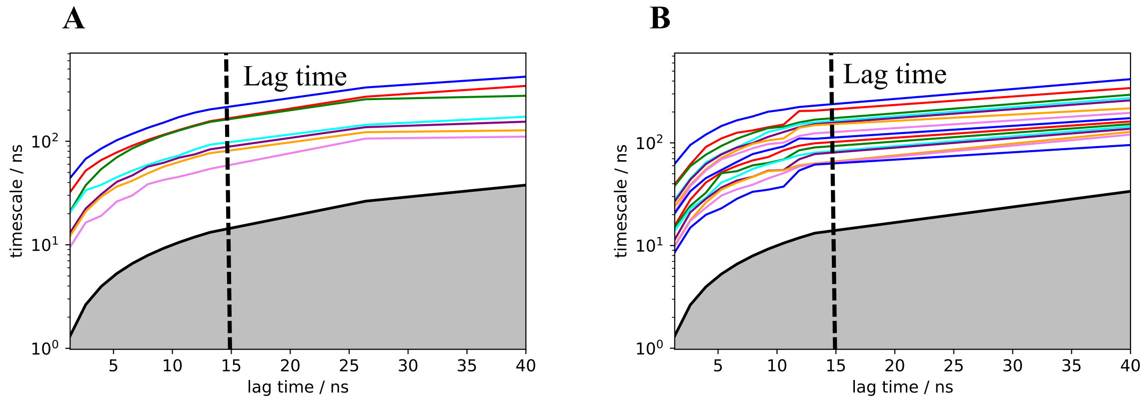

Figure S12. Determining lag-times for Hidden Markov Models (HMMs). The implied timescales associated with the HMMs for Aβ40 (A) and Aβ42 (B).The transition networks describing the dimerization process were constructed at a lag-time of 15 ns where the timescales appear converged.

REFERENCES AND NOTES

1. R. Tycko, Amyloid polymorphism: Structural basis and neurobiological relevance. *Neuron* **86**, 632–645 (2015).

2. A. Aguzzi, C. Haass, Games played by rogue proteins in prion disorders and alzheimer's disease. *Science* **302**, 814–818 (2003).

3. V. H. Finder, R. Glockshuber, Amyloid- aggregation. *Neurodegener Dis* **4**, 13–27 (2007).

4. T. E. Golde, S. Estus, L. H. Younkin, D. J. Selkoe, S. G. Younkin, Processing of the amyloid protein precursor to potentially amyloidogenic derivatives, *Science* **255**, 728–730 (1992).

5. Y. Miller, B. Ma, R. Nussinov, Polymorphism in Alzheimer Aβ amyloid organization reflects conformational selection in a rugged energy landscape. *Chem. Rev.* **110**, 4820–4838 (2010).

6. A. T. Petkova, R. D. Leapman, Z. Guo, W.-M. Yu, M. P. Mattson, R. Tycko, Self-propagating, molecular-level polymorphism in Alzheimer's β-amyloid fibrils. *Science* **307**, 262–265 (2005).

7. F. Meng, J. Yoo, H. S. Chung, Single-molecule fluorescence imaging and deep learning reveal highly heterogeneous aggregation of amyloid-β 42. *Proc. Natl. Acad. Sci. U.S.A.* **119**, e2116736119 (2022).

8. W. Qiang, W. M. Yau, J. X. Lu, J. Collinge, R. Tycko, Structural variation in amyloid-β fibrils from Alzheimer's disease clinical subtypes. *Nature* **541**, 217–221 (2017).

9. D. Thirumalai, G. Reddy, J. E. Straub, Role of water in protein aggregation and amyloid polymorphism. *Acc. Chem. Res.* **45**, 83–92 (2012).

10. J. A. Hardy, G. A. Higgins, Alzheimer's disease: The amyloid cascade hypothesis. *Science* **10**, 184–185 (1992).

11. D. J. Selkoe, Alzheimer's disease: A central role for amyloid. *J. Neuropathol. Exp. Neurol.* **53**, 438–447 (1994).

12. G. M. Shankar, S. Li, T. H. Mehta, A. Garcia-Munoz, N. E. Sheperdson, I. Smith, F. M. Brett, M. A. Farrell, M. J. Rowan, C. A. Lemere, C. M. Regan, D. M. Walsh, B. L. Sabatini, D. J. Selkoe, Amyloid-β protein dimers isolated directly from Alzheimer's brains impair synaptic plasticity and memory. *Nat. Med.* **14**, 837–842 (2008).

13. S. L. Bernstein, N. F. Dupuis, N. D. Lazo, T. Wyttenbach, M. M. Condron, G. Bitan, D. B. Teplow, J. Emm-Shea, B. T. Ruotolo, C. V. Robinson, M. T. Bowers, Amyloid-β protein oligomerization and the importance of tetramers and dodecamers in the aetiology of Alzheimer's disease. *Nat. Chem.* **1**, 326–331 (2009).

14. J. P. Cleary, D. M. Walsh, J. J. Hofmeister, G. M. Shankar, M. A. Kuskowski, D. J. Selkoe, K. H. Ashe, Natural oligomers of the amyloid-beta protein specifically disrupt cognitive function, *Nat. Neurosci.* **8**, 79–84 (2005).

15. S. B. Prusiner, Prions, *Proc. Natl. Acad. Sci. U.S.A.* **95**, 13363–13383 (1998).

16. W. P. Flavin, L. Bousset, Z. C. Green, Y. Chu, S. Skarpathiotis, M. J. Chaney, J. H. Kordower, R. Melki, E. M. Campbell, Endocytic vesicle rupture is a conserved mechanism of cellular invasion by amyloid proteins. *Acta Neuropathol.* **134**, 629–653 (2017).

17. J. E. Straub, D. Thirumalai, Toward a molecular theory of early and late events in monomer to amyloid fibril formation. *Annu. Rev. Phys. Chem.* **62**, 437–463 (2011).

18. B. Tarus, J. E. Straub, D. Thirumalai, Dynamics of Asp23−Lys28 salt-bridge formation in $A\beta_{10\text{-}35}$ monomers. *J. Am. Chem. Soc.* **128**, 16159–16168 (2006).

19. M. S. Li, N. T. Co, G. Reddy, C.-K. Hu, J. E. Straub, D. Thirumalai, Factors governing fibrillogenesis of polypeptide chains revealed by lattice models, *Phys. Rev. Lett.* **105**, 218101 (2010).

20. P. Zhurvalev, G. Reddy, J. E. Straub, D. Thirumalai, Propensity to form amyloid fibrils is encoded as excitations in the free energy landscape of monomeric proteins, *J. Mol. Biol.* **426**, 2653–2666 (2014).

21. D. Chakraborty, J. E. Straub, D. Thirumalai, Differences in the free energies between the excited states of Aβ40 and Aβ42 monomers encode their aggregation propensities. *Proc. Natl. Acad. Sci. U.S.A.* **117**, 19926–19937 (2020).

22. P. Neudecker, P. Robustelli, A. Cavalli, P. Walsh, P. Lundstrom, A. Zarrine-Afsar, S. Sharpe, M. Vendruscolo, L. E. Kay, Structure of an intermediate state in protein folding and aggregation. *Science* **336**, 362–366 (2012).

23. T. Kakeshpour, V. Ramanujam, C. A. Barnes, Y. Shen, J. Ying, A. Bax, A lowly populated, transient β-sheet structure in monomeric Aβ1-42 identified by multinuclear NMR of chemical denaturation. *Biophys. Chem.* **270**, 106531 (2021).

24. A. Kumar, D. Chakraborty, M. L. Mugnai, J. E. Straub, D. Thirumalai, Sequence determines the switch in the fibril forming regions in the low-complexity fus protein and its variants, *J. Phys. Chem. Lett.* **12**, 9026–9032 (2021).

25. J. Roche, Y. Shen, J. H. Lee, J. Ying, A. Bax, Monomeric $A\beta^{1-40}$ and $A\beta^{1-42}$ peptides in solution adopt very similar ramachandran map distributions that closely resemble random coil. *Biochemistry* **55**, 762–775 (2016).

26. K. A. Ball, A. H. Phillips, P. S. Nerenberg, N. L. Fawzi, D. E. Wemmer, T. Head-Gordon, Homogeneous and heterogeneous tertiary structure ensembles of amyloid-β peptides *Biochemistry* **50**, 7612–7628 (2011).

27. D. J. Rosenman, C. Wang, A. E. García, Characterization of Aβ monomers through the convergence of ensemble properties among simulations with multiple force fields. *J. Phys. Chem. B* **120**, 259–277 (2016).

28. Y. S. Lin, G. R. Bowman, K. A. Beauchamp, V. S. Pande, Investigating how peptide length and a pathogenic mutation modify the structural ensemble of amyloid beta monomer. *Biophys. J.* **102**, 315–324 (2012).

29. J. T. Jarrett, E. P. Berger, P. T. Lansbury Jr., The carboxy terminus of the beta amyloid protein is critical for the seeding of amyloid formation: Implications for the pathogenesis of Alzheimer's disease, *Biochemistry* **32**, 4693–4697 (1993).

30. G. Meisl, X. Yang, E. Hellstrand, B. Frohm, J. B. Kirkegaard, S. I. A. Cohen, C. M. Dobson, S. Linse, T. Knowles, Differences in nucleation behavior underlie the contrasting aggregation kinetics of the Aβ40 and Aβ42 peptides *Proc. Natl. Acad. Sci. U.S.A.* **111**, 9384–9389 (2014).

31. X. Yang, G. Meisl, B. Frohm, S. Linse, On the role of sidechain size and charge in the aggregation of aβ42 with familial mutations, *Proc. Natl. Acad. Sci. U.S.A.* **115**, E5849–E5858 (2018).

32. J.-X. Lu, W. Qiang, W.-M. Yau, C. D. Schwieters, S. C. Meredith, R. Tycko, Molecular structure of β-amyloid fibrils in Alzheimer's disease brain tissue. *Cell* **154**, 1257–1268 (2013).

33. M. A. Walti, F. Ravotti, H. Arai, C. G. Glabe, J. S. Wall, A. Bockmann, P. Guntert, B. H. Meier, R. Riek, Atomic-resolution structure of a disease-relevant Aβ(1–42) amyloid fibril, *Proc. Natl. Acad. Sci. U.S.A.* **1**, E4976–E4984 (2016).

34. F. Meng, M. M. Bellaiche, J. Y. Kim, G. H. Zerze, R. B. Best, H. S. Chung, Highly disordered amyloid-β monomer probed by single-molecule FRET and MD simulation. *Biophys. J.* **114**, 870–884 (2018).

35. N. Rezaei-Ghaleh, G. Parigi, M. Zweckstetter, Reorientational dynamics of amyloid-β from NMR spin relaxation and molecular simulation. *J. Phys. Chem. Lett.* **10**, 3369–3375 (2019).

36. T. Zhou, A. Calfisch, Distribution of reciprocal of interatomic distances: A fast structural metric. *J. Chem. Theory Comput.* **8**, 2930–2937 (2012).

37. S. V. Krivov, M. Karplus, Free energy disconnectivity graphs: Application to peptide models. *J. Chem. Phys.* **117**, 10894–10903 (2002).

38. L. Ford, D. R. Fulkerson, Maximal flow through a network. *Can. J. Math.* **8**, 399–404 (1956).

39. D. J. Wales, M. A. Miller, T. R. Walsh, Archetypal energy landscapes. *Nature* **394**, 758–760 (1998).

40. D. A. Evans, D. J. Wales, Free energy landscapes of model peptides and proteins. *J. Chem. Phys.* **118**, 3891–3897 (2003).

41. F. Noé, S. Fischer, Transition networks for modeling the kinetics of conformational change in macromolecules. *Curr. Opin. Struct. Biol.* **18**, 154–162 (2008).

42. B. Tarus, J. E. Straub, D. Thirumalai, Structures and free-energy landscapes of the wild type and mutants of the $A\beta_{21–30}$ peptide are determined by an interplay between intrapeptide electrostatic and hydrophobic interactions. *J. Mol. Biol.* **379**, 815–829 (2008).

43. S. H. Chong, S. Ham, Folding free energy landscape of ordered and intrinsically disordered proteins. *Sci. Rep.* **9**, 14927 (2019).

44. N. D. Lazo, M. A. Grant, M. C. Condron, A. C. Rigby, D. B. Teplow, On the nucleation of amyloid-β monomer folding. *Protein Sci.* **14**, 1581–1596 (2005).

45. N. L. Fawzi, A. H. Phillips, J. Z. Ruscio, M. Doucleff, D. E. Wemmer, T. Head-Gordon, Structure and dynamics of the $A\beta_{21-30}$ peptide from the interplay of NMR experiments and molecular simulations. *J. Am. Chem. Soc.* **130**, 6145–6158 (2008).

46. A. Baumketner, S. L. Bernstein, T. Wyttenbach, N. D. Lazo, D. B. Teplow, M. T. Bowers, J. Shea, Structure of the 21-30 fragment of amyloid β-protein. *Protein Sci.* **15**, 1239–1247 (2006).

47. B. Ma, R. Nussinov, Stabilities and conformations of Alzheimer's β-amyloid peptide oligomers ($A\beta_{16–22}$, $A\beta_{16–35}$, and $A\beta_{10–35}$): Sequence effects *Proc. Natl. Acad. Sci. U.S.A.* **99**, 14126–14131 (2002).

48. K. L. Sciaretta, D. J. Gordon, A. T. Petkova, R. Tycko, S. C. Meredith, Aβ40-lactam(D23/K28) models a conformation highly favorable for nucleation of amyloid. *Biochemistry* **44**, 6003–6014 (2005).

49. G. Reddy, J. E. Straub, D. Thirumalai, Influence of preformed Asp23−Lys28 salt bridge on the conformational fluctuations of monomers and dimers of Aβ peptides with implications for rates of fibril formation. *J. Phys. Chem. B* **113**, 1162–1172 (2009).

50. A. T. Petkova, Y. Ishi, J. J. Balbach, O. N. Antzutkin, R. D. Leapman, F. Delaglio, R. Tycko, A structural model for Alzheimer's β-amyloid fibrils based on experimental constraints from solid state NMR. *Proc. Natl. Acad. Sci. U.S.A.* **99**, 16742–16747 (2002).

51. B. Chandra, D. Bhowmik, B. K. Maity, K. R. Mote, D. Dhara, R. Venkatramani, S. Maiti, P. K. Madhu, Major reaction coordinates linking transient amyloid-β oligomers to fibrils measured at atomic level. *Biophys. J.* **113**, 805–816 (2017).

52. A. Korn, S. McLennan, J. Adler, M. Krueger, D. Surendran, S. Maiti, D. Huster, Amyloid β (1–40) toxicity depends on the molecular contact between phenylalanine 19 and leucine 34. *ACS Chem. Neurosci.* **9**, 790–799 (2018).

53. F. Hoffmann, J. Adler, B. Chandra, K. R. Mote, G. Bekcioglu-Neff, D. Sebastiani, D. Huster, Perturbation of the F19-L34 contact in amyloid β (1-40) fibrils induces only local structural changes but abolishes cytotoxicity. *J. Phys. Chem. Lett.* **8**, 4740–4745 (2017).

54. A. Potapov, W.-M. Yau, R. Ghirlando, K. R. Thurber, R. Tycko, Successive stages of amyloid-β self-assembly characterized by solid-state nuclear magnetic resonance with dynamic nuclear polarization. *J. Am. Chem. Soc.* **137**, 8294– (2015).

55. T. Luhrs, C. Ritter, M. Adrian, D. Riek-Loher, B. Bohrmann, H. Dobeli, D. Schubert, R. Riek, 3D structure of Alzheimer's amyloid-β(1–42) fibrils. *Proc. Natl. Acad. Sci. U.S.A.* **102**, 17342–17347 (2005).

56. M. T. Colvin, R. Silvers, Q. Z. Ni, T. V. Can, I. Sergeyev, M. Rosay, K. J. Donovan, B. Michael, J. Wall, S. Linse, R. G. Griffin, Atomic resolution structure of monomorphic $A\beta_{42}$ amyloid fibrils. *J. Am. Chem. Soc.* **138**, 9663–9674 (2016).

57. L. Gremer, D. Scholzel, C. Schenk, E. Reinartz, J. Labahn, R. B. G. Ravelli, M. Tusche, C. Lopez-Iglesias, W. Hoyer, H. Heise, D. Willbold, G. F. Schroder, Fibril structure of amyloid-β(1–42) by cryo–electron microscopy. *Science* **358**, 116–119 (2017).

58. Y. Yan, C. Wang, Aβ42 is more rigid than Aβ40 at the C terminus: Implications for Aβ aggregation and toxicity. *J. Mol. Biol.* **364**, 853–862 (2006).

59. N. G. V. Kempen, *Stochastic Processes in Physics and Chemistry* (Elsevier, 1992).

60. W. C. Swope, J. W. Pitera, F. Suits, Describing protein folding kinetics by molecular dynamics simulations. 1. Theory. *J. Phys. Chem. B* **108**, 6571–6581 (2004).

61. Y. Levy, O. Becker, Effect of conformational constraints on the topography of complex potential energy surfaces. *Phys. Rev. Lett.* **81**, 1126–1129 (1998).

62. U. R. Shrestha, P. Juneja, Q. Zhang, V. Gurumoorthy, J. M. Borreguero, V. Urban, X. Cheng, S. V. Pingali, J. C. Smith, H. M. O'Neill, L. Petridis, Generation of the configurational ensemble of an intrinsically disordered protein from unbiased molecular dynamics simulation. *Proc. Natl. Acad. Sci. U.S.A.* **116**, 20446–20452 (2019).

63. S. Acharya, K. P. Srivastava, S. Nagarajan, L. J. Lapidus, Monomer dynamics of alzheimer peptides and kinetic control of early aggregation in Alzheimer's disease. *ChemPhysChem* **17**, 3470–3479 (2016).

64. V. K. de Souza, J. D. Stevenson, S. P. Niblett, J. D. Farrell, D. J. Wales, Defining and quantifying frustration in the energy landscape: Applications to atomic and molecular clusters, biomolecules, jammed and glassy systems. *J. Chem. Phys.* **146**, 124103 (2017).

65. D. J. Wales, Energy landscapes: Some new horizons. *Curr. Opin. Struct. Biol.* **20**, 3–10 (2010).

66. D. J. Wales, Calculating rate constants and committor probabilities for transition networks by graph transformation. *J. Chem. Phys.* **130**, 204111 (2009).

67. F. Noe, H. Wu, J.-H. Prinz, N. Plattner, Projected and hidden markov models for calculating kinetics and metastable states of complex molecules. *J. Chem. Phys.* **139**, 184114 (2013).

68. P. H. Nguyen, M. S. Li, G. Stock, J. E. Straub, D. Thirumalai, Monomer adds to preformed structured oligomers of Aβ-peptides by a two-stage dock–lock mechanism. *Proc. Natl. Acad. Sci. U.S.A.* **104**, 111–116 (2007).

69. B. Efron, C. Stein, The Jackknife estimate of variance. *Ann. Statist.* **9**, 586–596 (1981).

70. D. J. Wales, Dynamical signatures of multifunnel energy landscapes. *J. Phys. Chem. Lett.* **13**, 6349–6358 (2022).

71. A. Levin, T. O. Mason, L. Adler-Abramovich, A. K. Buell, G. Meisl, C. Galvagnion, Y. Bram, S. A. Stratford, C. M. Dobson, T. J. Knowles, E. Gazit, Ostwald's rule of stages governs structural transitions and morphology of dipeptide supramolecular polymers. *Nat. Commun.* **13**, 5219 (2014).

72. A. Paul, S. Samantray, M. Anteghini, M. Khaled, B. Strodel, Thermodynamics and kinetics of the amyloid-β peptide revealed by Markov state models based on MD data in agreement with experiment. *Chem. Sci.* **12**, 6652–6669 (2021).

73. F. C. Zegarra, D. Homouz, Y. Eliaz, A. G. Gasic, M. S. Cheung, Impact of hydrodynamic interactions on protein folding rates depends on temperature. *Phys. Rev. E* **97**, 032402 (2018).

74. Y. Goldtzvik, Z. Zhang, D. Thirumalai, Importance of hydrodynamic interactions in the stepping kinetics of kinesin. *J. Phys. Chem. B* **120**, 2071–2075 (2016).

75. A. K. Paravastu, R. D. Leapman, W. M. Yau, R. Tycko, Molecular structural basis for polymorphism in Alzheimer's β-amyloid fibrils. *Proc. Natl. Acad. Sci. U.S.A.* **105**, 18349–18354 (2008).

76. M. Kollmer, W. Close, L. Funk, J. Rasmussen, A. Bsoul, A. Schierhorn, M. Schmidt, C. J. Sigurdson, M. Jucker, M. Fändrich, Cryo-EM structure and polymorphism of Aβ amyloid fibrils purified from Alzheimer's brain tissue. *Nat. Commun.* **10**, 4760 (2019).

77. U. Ghosh, K. R. Thurber, W. M. Yau, R. Tycko, Molecular structure of a prevalent amyloid-β fibril polymorph from Alzheimer's disease brain tissue. *Proc. Natl. Acad. Sci. U.S.A.* **118**, e2023089118 (2021).

78. A. M. Stern, Y. Yang, A. L. Meunier, W. Liu, Y. Cai, M. Ericsson, L. Liu, M. Goedert, S. H. W. Scheres, D. J. Selkoe, Abundant Aβ fibrils in ultracentrifugal supernatants of aqueous extracts from Alzheimer's disease brains. *bioRxiv* 2022.10.18.512754 (2022).

79. Y. Yang, D. Arseni, W. Zhang, M. Huang, S. Lovestam, M. Schweighauser, A. Kotecha, A. G. Murzin, S. Y. Peak-Chew, J. Macdonald, I. Lavenir, H. J. Garringer, E. Gelpi, K. L. Newell, G. G. Kovacs, R. Vidal, B. Ghetti, B. Ryskeldi-Falcon, S. H. W. Scheres, M. Goedert, Cryo-em structures of amyloid-β 42 filaments from human brains. *Science* **375**, 167–172 (2022).

80. M. Schmidt, A. Rohou, J. K. Yadav, C. Schiene-Fischer, M. Fandrich, N. Grigorieff, Peptide dimer structure in an Aβ(1–42) fibril visualized with cryo-em. *Proc. Natl. Acad. Sci. U.S.A.* **112**, 11858–11863 (2015).

81. D. Granata, F. Baftizadeh, J. Habchi, C. Galvagnion, A. De Simone, C. Camilloni, A. Laio, M. Vendruscolo, The inverted free energy landscape of an intrinsically disordered peptide by simulations and experiments, *Sci. Rep.* **5**, 15449 (2015).

82. H. Kang, B. Luan, R. Zhou, Glassy dynamics in mutant huntingtin proteins. *J. Chem. Phys.* **149**, 072333 (2018).

83. I. L. Morgan, R. Avinery, G. Rahamim, R. Beck, O. A. Saleh, Glassy dynamics and memory effects in an intrinsically disordered protein construct. *Phys. Rev. Lett.* **125**, 058001 (2020).

84. Y. Chebaro, A. J. Ballard, D. Chakraborty, D. J. Wales, Intrinsically disordered energy landscapes. *Sci. Rep.* **5**, 10386 (2015).

85. B. Strodel, C. S. Whittleston, D. J. Wales, Thermodynamics and kinetics of aggregation for the gnnqqny peptide, *J. Am. Chem. Soc.* **129**, 16005–16014 (2007).

86. Y. Lu, P. Derreumaux, Z. Guo, N. Mousseau, G. Wei, Thermodynamics and dynamics of amyloid peptide oligomerization are sequence dependent. *Proteins* **75**, 954–963 (2009).

87. R. Zwanzig, Diffusion in a rough potential. *Proc. Natl. Acad. Sci. U.S.A.* **85**, 2029–2030 (1988).

88. D. T. Murray, M. Kato, Y. Lin, K. R. Thurber, I. Hung, S. L. McKnight, R. Tycko, Structure of fus protein fibrils and its relevance to self-assembly and phase separation of low-complexity domains, *Cell* **171**, 615–627.e16 (2017).

89. M. Lee, U. Ghosh, K. R. Thurber, M. Kato, R. Tycko, Molecular structure and interactions within amyloid-like fibrils formed by a low-complexity protein sequence from fus. *Nat. Commun.* **11**, 5735 (2020).

90. M. Kato, S. L. Mcknight, Extrasynaptic acetylcholine signaling through a muscarinic receptor regulates cell migration. *Proc. Natl. Acad. Sci. U.S.A.* **118**, e1904338118 (2021).

91. S. A. Kotler, V. Tugarinov, T. Schmidt, G. M. Clore, Probing initial transient oligomerization events facilitating huntingtin fibril nucleation at atomic resolution by relaxation-based nmr, *Proc. Natl. Acad. Sci. U.S.A.* **116**, 3562–3571 (2019).

92. T. R. Jahn, M. J. Parker, S. W. Homans, S. E. Radford, Amyloid formation under physiological conditions proceeds via a native-like folding intermediate. *Nat. Struct. Mol. Biol.* **13**, 195–201 (2006).

93. D. C. Latshaw, M. Cheon, C. K. Hall, Effects of macromolecular crowding on amyloid beta (16-22) aggregation using coarse-grained simulations. *J. Phys. Chem. B* **118**, 13513–13526 (2014).

94. H. Fatafta, M. Khaled, M. C. Owen, B. Strodel, Amyloid-β peptide dimers undergo a random coil to β-sheet transition in the aqueous phase but not at the neuronal membrane. *Proc. Natl. Acad. Sci. U.S.A.* **118**, e2106210118 (2021).

95. U. Baul, D. Chakraborty, M. L. Mugnai, J. E. Straub, D. Thirumalai, Sequence effects on size, shape, and structural heterogeneity in intrinsically disordered proteins. *J. Phys. Chem. B* **123**, 3462–3474 (2019).

96. M. R. Betancourt, D. Thirumalai, Pair potentials for protein folding: Choice of reference states and sensitivity of predicted native states to variations in the interaction schemes. *Protein Sci.* **8**, 361–369 (1999).

97. D. Ermak, J. A. McCammon, Brownian dynamics with hydrodynamic interactions, *J. Chem. Phys.* **69**, 1352–1360 (1978).

98. J. Rotne, S. Prager, Variational treatment of hydrodynamic interactions in polymers. *J. Chem. Phys.* **50**, 4831–4837 (1969).

99. P. Zuk, E. Wajnryb, K. A. Mizerski, P. Szymczak, Rotne-prager-yamakawa approximation for different-sized particles in application to macromolecular bead models. *J. Fluid Mech.* **741**, R5 (2014).

100. M. K. Scherer, B. Trendelkamp-Schroer, F. Paul, G. Perez-Hernandez, M. Hoffmann, N. Plattner, C. Wehmeyer, J.-H. Prinz, F. Noe, PyEMMA 2: A software package for estimation, validation, and analysis of Markov models. *J. Chem. Theory Comput.* **11**, 5525–5542 (2015).

101. D. J. Wales, *Energy Landscapes* (Cambridge Univ. Press, 2003).

102. S. V. Krivov, M. Karplus, Hidden complexity of free energy surfaces for peptide (protein) folding. *Proc. Natl. Acad. Sci. U.S.A.* **101**, 14766–14770 (2004).

103. R. E. Gomory, T. C. Hu, Multi-terminal network flows, *J. Soc. Indust. Appl. Math.* **9**, 551–570 (1961).

104. D. J. Wales, PATHSAMPLE: A program for generating connected stationary point databases and extracting global kinetics; www-wales.ch.cam.ac.uk/software.html.

105. J. A. Joseph, K. Roder, D. Chakraborty, R. G. Mantell, D. J. Wales, Exploring biomolecular energy landscapes, *Chem. Commun.* **53**, 6974–6988 (2017).

106. M. Miller, D. J. Wales, V. de Souza, disconnectionDPS; www-wales.ch.cam.ac.uk/software.html.

107. D. Chakraborty, D. J. Wales, A multifunnel energy landscape encodes the competing α-helix and β-hairpin conformations for a designed peptide. *Phys. Chem. Chem. Phys.* **22**, 1359–1370 (2020).

108. K. Röder, D. J. Wales, Evolved Minimal Frustration in Multifunctional Biomolecules. *J. Phys. Chem. B* **122**, 10989–10995 (2018).

109. D. Gront, S. Kmiecik, A. Kolinski, Backbone building from quadrilaterals: A fast and accurate algorithm for protein backbone reconstruction from alpha carbon coordinates. *J. Comput. Chem.* **128**, 1593–1597 (2007).

110. G. G. Krivov, M. V. Shapalov, R. L. Dunbrack, Improved prediction of protein side-chain conformations with SCWRL4. *Proteins* **77**, 778–795 (2009).

111. A. Onufriev, D. Bashford, D. A. Case, Modification of the generalized born model suitable for macromolecules. *J. Phys. Chem. B* **104**, 3712–3720 (2000).

112. D. A. Case, T. A. Darden, T. Cheatham, C. L. Simmerling, J. Wang, R. E. Duke, R. Luo, R. C. Walker, W. Zhang, K. M. Merz, B. Roberts, S. Hayik, A. Roitberg, G. Seabra, J. Swails, A. W. Goetz, I. Kolossváry, Amber 12 (2012); http://ambermd.org/.
